# Machine learning identifies stroke features between species

**DOI:** 10.7150/thno.51887

**Published:** 2021-01-01

**Authors:** Salvador Castaneda-Vega, Prateek Katiyar, Francesca Russo, Kristin Patzwaldt, Luisa Schnabel, Sarah Mathes, Johann-Martin Hempel, Ursula Kohlhofer, Irene Gonzalez-Menendez, Leticia Quintanilla-Martinez, Ulf Ziemann, Christian la Fougere, Ulrike Ernemann, Bernd J. Pichler, Jonathan A. Disselhorst, Sven Poli

**Affiliations:** 1 Werner Siemens Imaging Center, Department of Preclinical Imaging and Radiopharmacy, Eberhard Karls University Tuebingen, Tuebingen, Germany.; 2 Department of Nuclear Medicine and Clinical Molecular Imaging, Eberhard Karls University Tuebingen, Tuebingen, Germany.; 3 Max Planck Institute for Intelligent Systems, Tuebingen, Germany.; 4 Department of Neurology & Stroke, and Hertie Institute for Clinical Brain Research, Eberhard Karls University Tuebingen, Tuebingen, Germany.; 5 Department of Diagnostic and Interventional Neuroradiology, Eberhard Karls University Tuebingen, Tuebingen, Germany.; 6 Institute of Pathology and Neuropathology and Comprehensive Cancer Center Tübingen, Eberhard Karls University Tuebingen, Tübingen, Germany.

**Keywords:** Ischemic stroke, Translational medicine, Machine learning, Stroke segmentation Neuroimaging

## Abstract

Identification and localization of ischemic stroke (IS) lesions is routinely performed to confirm diagnosis, assess stroke severity, predict disability and plan rehabilitation strategies using magnetic resonance imaging (MRI). In basic research, stroke lesion segmentation is necessary to study complex peri-infarction tissue changes. Moreover, final stroke volume is a critical outcome evaluated in clinical and preclinical experiments to determine therapy or intervention success. Manual segmentations are performed but they require a specialized skill set, are prone to inter-observer variation, are not entirely objective and are often not supported by histology. The task is even more challenging when dealing with large multi-center datasets, multiple experimenters or large animal cohorts. On the other hand, current automatized segmentation approaches often lack histological validation, are not entirely user independent, are often based on single parameters, or in the case of complex machine learning methods, require vast training datasets and are prone to a lack of model interpretation.

**Methods:** We induced IS using the middle cerebral artery occlusion model on two rat cohorts. We acquired apparent diffusion coefficient (ADC) and T2-weighted (T2W) images at 24 h and 1-week after IS induction. Subsets of the animals at 24 h and 1-week post IS were evaluated using histology and immunohistochemistry. Using a Gaussian mixture model, we segmented voxel-wise interactions between ADC and T2W parameters at 24 h using one of the rat cohorts. We then used these segmentation results to train a random forest classifier, which we applied to the second rat cohort. The algorithms' stroke segmentations were compared to manual stroke delineations, T2W and ADC thresholding methods and the final stroke segmentation at 1-week. Volume correlations to histology were also performed for every segmentation method. Metrics of success were calculated with respect to the final stroke volume. Finally, the trained random forest classifier was tested on a human dataset with a similar temporal stroke on-set. Manual segmentations, ADC and T2W thresholds were again used to evaluate and perform comparisons with the proposed algorithms' output.

**Results:** In preclinical rat data our framework significantly outperformed commonly applied automatized thresholding approaches and segmented stroke regions similarly to manual delineation. The framework predicted the localization of final stroke regions in 1-week post-stroke MRI with a median Dice similarity coefficient of 0.86, Matthew's correlation coefficient of 0.80 and false positive rate of 0.04. The predicted stroke volumes also strongly correlated with final histological stroke regions (Pearson correlation = 0.88, P < 0.0001). Lastly, the stroke region characteristics identified by our framework in rats also identified stroke lesions in human brains, largely outperforming thresholding approaches in stroke volume prediction (P<0.01).

**Conclusion:** Our findings reveal that the segmentation produced by our proposed framework using 24 h MRI rat data strongly correlated with the final stroke volume, denoting a predictive effect. In addition, we show for the first time that the stroke imaging features can be directly translated between species, allowing identification of acute stroke in humans using the model trained on animal data. This discovery reduces the gap between the clinical and preclinical fields, unveiling a novel approach to directly co-analyze clinical and preclinical data. Such methods can provide further biological insights into human stroke and highlight the differences between species in order to help improve the experimental setups and animal models of the disease.

## Introduction

Ischemic stroke (IS) is a debilitating disease with high morbidity and mortality. The American Heart Association estimates that by the year 2030 there will be an additional 3.4 million adults developing stroke in the United States; a projected increment of 20.5% from 2012 1. Accurate determination of stroke location is fundamental for understanding the processes of neuroplasticity and neurovascular changes occurring in the stroke and peri-infarct region 2. At the same time stroke segmentation is of high clinical relevance for developing and planning of rehabilitation therapies 3,4, predicting patient outcome and disabilities 5-7, and personalizing novel neuromodulation approaches 5,8-11. In order to perform IS segmentation the neuroimaging field has relied on Magnetic Resonance Imaging (MRI), where T2-weighted (T2W) images and diffusion weighted images are the most routinely applied sequences.

T2W images measure the nuclear magnetic resonance transverse relaxation time in tissue, and in the case of stroke, depict the widely understood vasogenic edema. The blood brain barrier presents massive breakage and permeability, which increases water content, causing tissue T2-relaxivity changes allowing edema identification 12. T2W signal intensity has been described to increase in the stroke area as early as 3.5 h after stroke 13 and has been thoroughly documented, and therefore regularly used to assess stroke location and size of the lesion 12-17. However, manual contouring of T2W images to segment stroke lesions 24 h post-stroke have found that edema significantly overestimates the stroke volume 18. One week after stroke, the T2W signal intensity reduces and a more accurate delimitation of the stroke can be performed 12,19,20. Hence, in humans final stroke volume has been defined as early as 1-week post-stroke using MRI 19,21. Unfortunately, MRI scans 1-week post-stroke are seldom produced since they are not part of the clinical routine, so the final stroke volume is not often evaluated.

Diffusion weighted images are used to calculate apparent diffusion coefficient (ADC) maps, which indicate water diffusion restrictions in the stroke tissue due to cellular swelling, otherwise referred to as cytotoxic edema 22-24. ADC decreases in the stroke area as early as 30 min after occlusion 25. Maximum ADC reductions occur from 12 h to 24 h after stroke onset and normalize to baseline values after 1 week with subsequent increment over baseline values as necrosis ensues in the chronic phase 20,26 (increased ADC due to low cellularity). ADC interpretation is dynamic and complex and it has been associated to microstructural tissue changes involving microvasculature and also blood brain barrier permeability 27-29. In stroke, reduced ADC is considered a strong predictor of final stroke volume 15,30,31. ADC reductions correlate to 2,3,5-Triphenyltetrazolium chloride (TTC) staining, and hematoxylin and eosin (H&E) histology in the preclinical setting and to neurological deficits in patients 32-34. Therefore, ADC is a very relevant parameter for stroke segmentation, and therefore is routinely evaluated in clinical trials and diagnostics.

The joint analysis of ADC and T2W data can potentially provide information about combined lesion changes to more accurately segment final stroke volume. These parameters measure partly overlapping and distinct microstructural lesion traits, which in turn reflect different histological characteristics in the stroke region over time 13,26,35. High T2W signal intensity occurs at the same time as peak ADC reduction occurs in the first days after stroke onset 12,14,36. However, both ADC and T2W signal intensity are heterogenous in the stroke tissue, which is reflected in histology as necrosis, apoptosis, edema and initial neuroinflammatory processes 35,37-39. These processes are complex and often not redundant 14 making tissue segmentation difficult. Importantly however, the patterns of ADC and T2W during ischemic stroke are, for the most part, similar between mice, rats and humans 13,17,40-43.

Stroke lesion segmentation is often performed by experimentors using manual delineation of lesions. However, manual segmentations require a highly specialized skill set, are prone to inter-observer variation, are not entirely objective and quite often are not based on histological findings 44. The task is even more challenging when dealing with large multi-center datasets or large animal cohorts. Indeed, basic research and clinical trials require objective and reliable tools for stroke segmentation, which can be provided by automated image analyses 45,46. Segmentation models based on T2W and ADC have been shown to identify stroke regions in rats 46 and humans 22 separately. Thresholding of the parameters in the stroke areas is routinely performed on preclinical datasets, where the stroke region is modeled in a predetermined brain hemisphere 41,47,48. This approach is not always applicable on clinical data, where stroke lesions occurs in unpredictable and sometimes disseminated patterns. Several approaches have been proposed to accurately distinguish between healthy and ischemic tissue using complex machine learning (ML) algorithms 17,49-51. However, these models trained on human MRI-data lack histological validation, since there is no clinical indication to obtain histology from stroke patients to validate the diagnosis. Moreover, they are not entirely user independent, are often based on single parameters, or in the case of complex machine learning methods, require vast training datasets with numerous imaging parameters prone to a lack of model interpretation 49,50,52-54.

In this report, we propose a histologically validated ML framework using combined ADC and T2W imaging data from rats using the well-known middle cerebral artery occlusion (MCAO) stroke model 55. Our aim was to accurately segment stroke volumes in animals and compare the automated segmentation to commonly used thresholding approaches. Moreover, we show for the first time the feasibility of using a ML model trained on rat-brain data to identify stroke regions in humans.

## Materials and Methods

### Animal experiments

The rat experiments were approved by the local ethics committee (Regierungspraesidium Tuebingen, N3/13). Male Sprague Dawley rats (Charles Rivers Laboratories, Germany) were housed in individual ventilated cages with standard wood-chip bedding under controlled temperature and humidity. Stroke surgery following a reversible (100 min) MCAO stroke model was performed in all animals (please see supplementary materials for further details). The rats were divided into Training (n = 12) and Testing group (n = 20). The animals originally took part in a preclinical trial, where the Testing group received an unsuccessful treatment therapy, while the Training group did not receive any form of therapy. Details regarding the conditions of the trial can be found in the supplementary material. An overview of the imaging and post-processing pipeline can be seen in supplementary figure S1 and the animal numbers for all experiments are detailed in supplementary Table S1.

### Preclinical Imaging - Acquisitions

MRI was performed using a 7 Tesla ClinScan small animal scanner using a rat whole body transmitter coil and a 4-channel rat brain surface receiving coil (Bruker BioSpin, Germany). The imaging protocol consisted of T2W and diffusion weighted acquisitions. ADC maps were calculated from the diffusion images. All animals were scanned using MRI 24 h post induction of IS. A subset of animals was followed-up with MRI 1-week post stroke induction (n = 9) using the same imaging protocols. Additional details of imaging protocols and post-processing are provided in the supplementary material.

### Clinical Imaging - Case selection and Acquisitions

Human brain scans were retrospectively evaluated following institutional ethical guidelines under ethical approval number 912/2018BO2. We evaluated a total of 12 patients that were scanned with MRI due to a clinical suspicion of IS, which had not undergone clinical therapies for stroke (e.g. thrombolysis). MRI scans were produced after a stroke-symptom onset of 6 to 24 h (e.g. speech impediment, paresis and paresthesia). Four patients presented no radiological signs of stroke, as they were studied under suspicion of transient ischemic attacks (TIA). The eight remaining patients presented IS lesions in MRI. From the eight stroke patients, four were followed up with MRI after 1-week. Scans were performed in a 1.5 Tesla MRI scanner (Magnetom Espree, Siemens Healthineers) using a routine clinical protocol including T2W images and diffusion weighted images followed by ADC map calculations. Further information is provided in supplementary material.

### Machine learning

Data evaluation followed the structured pipeline detailed in supplementary figure S1. In order to identify stroke features, we segmented the preclinical imaging data of the Training group. The segmentation was performed by fitting a Gaussian Mixture Model (GMM) on the two-dimensional MRI data of rats comprising ADC and T2W image intensity values. For all animals in the Training group, the GMM clustering resulted in the labeling of image voxels as stroke and non-stroke. Subsequently, we used the voxel-level GMM labels and the collective imaging data of all Training group rats-with each voxel treated as an observation with two features-to train a Random Forest Classifier (RFC). The trained classifier was tested on the data from animals independent to the Training group, as well as on the human-brain data. Further details about the individual steps are provided in the sub-sections below.

### Clustering Training rats

Prior to clustering, the whole-brain pre-processed image data of all rats were combined to obtain a population dataset. As distance-based clustering algorithms are susceptible to scale differences between features, the population dataset was normalized to zero mean and unit standard deviation. Thereafter, to identify the cluster bearing stroke characteristics (reduced ADC and increased T2W signal intensity), only the voxels from the Training group were fitted using a GMM with varying number of mixture components 56. For each fit with 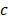 components, the stroke cluster (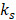) was selected as following:


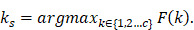


Where, 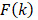
is calculated as:


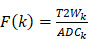


Here, 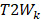 and 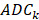 are the average values of the respective un-normalized parameters from the Training group for the component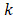. In addition, after each fit, the contralateral stroke fraction was calculated based on the total number of contralateral voxels present within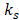. Finally, the optimal number of mixture components that resulted in a small contralateral stroke fraction was determined. This assumption is based on the histological confirmation that the contralateral hemisphere was not affected by the stroke-induction procedure, and thus, can be regarded as negative stroke lesion control. We found five mixture components to be optimal. Any further increase in the number of mixture components subdivided the stroke cluster without any significant reduction in the contralateral stroke fraction at the cost of increased model complexity, as shown in supplementary materials (supplementary figure S2).

### Classifier training

The population-based stroke cluster identified in the previous step served as a reference to detect the volume of acute stroke in the testing group rats. To obtain objective and repetitive evaluations of acute stroke volume, we trained an RFC using the combined data of all Training group rats recorded at the 24h timepoint. The associated training labels were a result of the GMM with optimal number of mixture components. Specifically, to simplify the supervised learning formulation from multi-class to a two-class classification problem, voxels from all the components other than 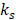 were regarded as non-stroke, irrespective of their biological characteristics. The classifier was trained using the un-normalized Training dataset. A total of 100 trees were used in training the model and both features were sampled for each decision split. In addition, the minimum number of observations per tree leaf (MinLeafSize parameter) was set to 1. The remaining parameters were kept to MATLAB's defaults.

### Testing - Detection of acute stroke in rat treatment groups

We applied the trained model on the data (with un-normalized T2W and ADC values) from the testing group. The classifier resulted in an acute stroke probability map for each rat, indicating the likelihood for each voxel to be classified as stroke.

### Thresholding preclinical data and region of interest evaluations

To make qualitative comparisons of the proposed ML framework to other relatively simple automated approaches, stroke volumes for rats in the testing group were also determined by thresholding the T2W images and ADC maps as has been performed by several authors 41,47,48. Based on these studies, the T2W stroke threshold was set to 125% of the mean value of the contralateral hemisphere, whereas a fixed value of 680×10^-6^ mm^2^/s was used to threshold acute stroke lesions in the ADC maps. The thresholding resulted in a lesion classification map that indicated whether each voxel is stroke or non-stroke.

The preclinical ML results were additionally compared to commonly performed evaluations consisting of manually drawn regions of interests (ROIs) on the aligned ADC and T2W images to delineate the stroke volume at 24 h (Manual ROI) without the aid of histology. The ROIs were drawn on all animals in consensus by two experienced users who were blinded to the animal group classification. The users also performed ROI analyses on the subset of animals belonging to all groups that were longitudinally imaged at the 1-week timepoint post stroke induction using the combination of ADC, T2W images, histology and immunohistochemistry. This final assessment was performed to allow a reduction of brain edema and obtain a more accurate segmentation of the final stroke volume. The ROI produced by using the 1-week post stroke induction ADC and T2W images is referred to as the Estimated Ground Truth (EGT) volume, since this measurement has been found to correlate with the final stroke volume in humans using MRI 19-21. We refer to this as EGT because it is only partially validated, since H&E histology was not performed on the whole stroke volume. The most accurate representation of ground truth stroke volume corresponds specifically to the selected MRI slices for which there exists a histological validation; therefore, only histologically validated MRI slices were used for the calculation of prediction metrics detailed in the Statistical Analysis section for rats. Images of the different segmentation approaches are compiled as joint probability maps to help visualize the average (across rats imaged at the 1-week timepoint) stroke volume; further details in supplementary materials.

### Histology and immunohistochemistry

Histological evaluations were performed on subsets of animals at 24 h (n = 9) and at 1-week (n = 20) post IS induction by a certified Pathologist. Briefly, after imaging, animals were intracardially perfused with 40 mL of phosphate buffered saline solution and 40 mL of 4% formaldehyde. The brains were dissected and further fixed, dehydrated and paraffin embedded. For histology, transversal sections 3 to 5 µm thick were cut on the brain regions with the visibly broadest stroke areas and stained using H&E. The certified pathologist evaluated all the histological slides and defined ROIs. Stroke areas were drawn on H&E slides using NDP.view software (Version 2.3.1, Hamamatsu Photonics K.K., Systems Division, Hamamatsu, Japan). As previously described, the H&E ROIs were directly compared with the corresponding MRI slice per animal in order to define the ground truth. Further information can be found in supplementary material.

### Testing - Detection of acute stroke in humans

The clinical dataset testing underwent the same evaluation as the rat dataset with only one minor difference. The T2W image for each patient was normalized using the instance mean and the standard deviation. We did this for three reasons: first, two different magnetic field strengths will alter the transversal relaxation time of water differently, making their direct comparison unfeasible. Second, the mean and standard deviation of the patient population were not applicable, because of the ambiguity in the exact stroke onset time point of each patient, which should also influence the T2W signal intensity. Third, T2W images are not quantitative, so normalizing can standardize the signal intensity obtained from each image dataset and allow a comparison between subjects. For testing on the clinical data, we trained another classifier using the un-normalized ADC and the normalized T2W population data of the Training group rats recorded at 24h timepoint. Moreover, the same ADC and T2 threshold segmentations method described above for rats was used to produce lesion classification maps on the human dataset. Metrics of success were then calculated for all segmentation approaches (described in statistical analysis).

### Clinical assessment

Two board-certified radiologists evaluated both ADC and T2W images of the twelve patients to identify and draw ROIs on stroke regions fitting the 24 h post-occlusion characteristics. The follow-up 1-week scans were also evaluated. The ROI intersection between both radiologists was used for both time points, the 24 h stroke ROI is referred as “Manual” delineation and the 1-week ROI as “EGT” analogous to the rat segmentation. The readers were blinded to previous clinical conditions, severity or localization of the lesions. These ROIs were delineated on the post-processed MRI data. After identification of the affected stroke hemisphere, thresholding was performed on ADC and T2W images as previously described on the rat datasets.

### Statistical analysis

One-way analysis of variance (ANOVA) was performed to test main group effects followed by Bonferroni correction for multiple group comparisons. ANOVA results are shown in the standard format with degrees of freedom, F-test and p-value. Student's t-tests were used for single comparisons where applicable at an alpha level of 0.05 and 0.95 confidence interval. Pearson's correlation coefficient was calculated and presented with the corresponding p-value per calculation. Additionally, we calculated a confusion matrix of the most commonly used metrics to evaluate the performance of the segmentation methods in both animals and humans. The metrics calculated consisted of accuracy, sensitivity, specificity, false discovery rate, false positive rate, positive predictive value and negative predictive value. Moreover, the Dice similarity coefficient and accuracy were calculated to evaluate diagnostic prediction 57. The mathematical expressions of these measures are provided in supplementary materials. Finally, we calculated the more conservative Matthew's Correlation Coefficient, since it has been suggested to provide a balanced and comprehensive account of the ratios that conform the confusion matrix 58-61. Differences between segmentation approaches with every metric were statistically evaluated using ANOVA. Confidence intervals for these experiments are provided in supplementary Tables S2-3. All statistical analyses were performed using MATLAB.

## Results

We first evaluate the rat brain dataset using the proposed ML framework. The calculated stroke segmentations predictions are then further analyzed against histological outcomes. Furthermore, we measure the algorithm's success on preclinical test data using common and robust prediction metrics. Thereafter, we apply the trained algorithm on human datasets and evaluate the prediction success using the same prediction metrics.

### Identification of stroke clusters in the rat brain

**Fig 1:**
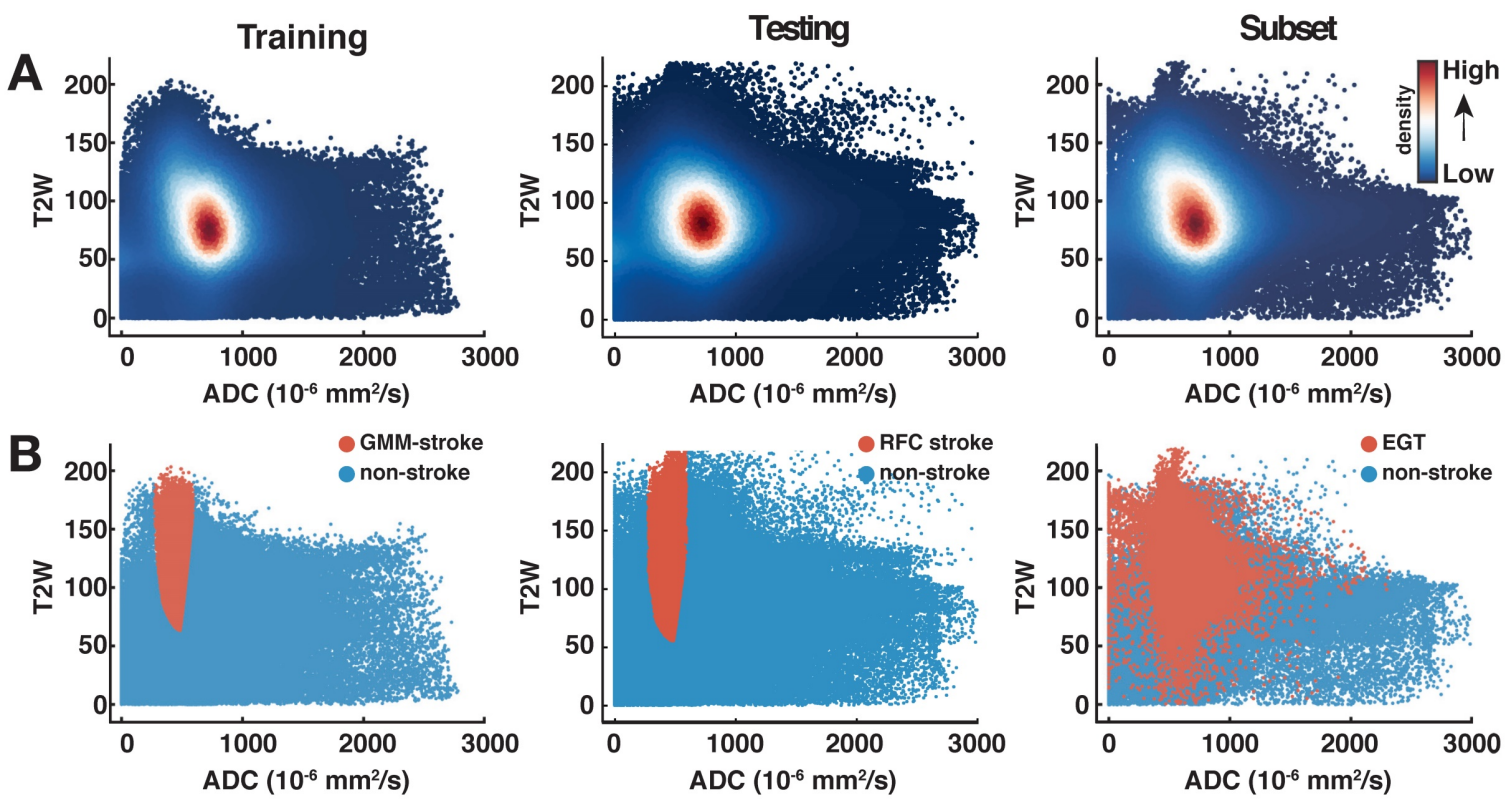
** Scatterplots of all animals per group. (A).** The density scatter plots show the distribution of all the rat brain data and the concentration of the voxels as a heat map. The distribution of apparent diffusion coefficient (ADC) and T2-weighted (T2W) image brain data of both animal groups is shown from left to right. The rightmost plot shows the distribution of the 24 h data belonging to the subset of animals (n=9) that underwent follow-up scans at the 1-week time point. Most data points are distributed in a similar range among all groups as can be seen by the spatial distribution of the highest density regions. **(B).** On the same distribution as above (panel A), the orange datapoints show for the Training animals the stroke cluster produced by the optimal Gaussian Mixture Model, whereas for the Testing group the stroke labeled predictions made by the Random Forest Classifier. The distribution on the rightmost plot shows the spread of the estimated ground truth (EGT) labels obtained from 1-week MRI. This comparison shows that the stroke labeled voxels predicted by our machine learning workflow are mostly located within the range of data of the EGT.

The distribution of preclinical T2W and ADC data for all animals in the two groups can be observed in the scatter plots of Fig. 1. Density scatter plots (A, top row) show the distribution of the acquired data from left to right for the training and testing groups. In the same row (Fig. 1A), the rightmost scatter plot shows the distribution of 24 h data of the subset of animals that received an MRI after 1-week. Most ADC voxels are found roughly within values between 500 to 1,100 x 10^-6^ mm^2^/s. The T2W data shows most voxels approximately between 50 to 125 (arbitrary units). This 2-dimensional (ADC and T2W) distribution of data is key to the identification of the stroke cluster. The training group data observed in the scatterplot of Fig. 1B, leftmost column, shows the stroke (orange) and non-stroke (blue) clusters identified using the optimal GMM. The stroke cluster has a relatively high T2W signal intensity in comparison to the rest of the voxels. At the same time, the ADC values of the stroke voxels lie roughly below 600 x 10^-6^ mm^2^/s. Similar characteristics can be observed for the testing group scatter plot. The rightmost scatter plot (Subset, Fig. 1B) shows in orange the MRI-based EGT stroke labels of the 1-week follow-up scans (supplementary Table S1). The visual comparison of the scatter plots shows how the training and testing groups produce stroke labels centered in the range of the EGT labels.

As expected, stroke lesions at 24 h show reduced ADC and hyperintense T2W. This interaction allows a precise identification of the stroke as demonstrated by the individual exemplary rat brain images in supplementary figure S3. Here, the pattern of ADC and T2W datapoints from Fig. 1 corresponding to the exemplary rats are reconstructed back into anatomical space as individual ML probability maps. Further information can be found in supplementary material.

### Quantification of whole stroke volumes using common approaches

The quantification of whole stroke volumes obtained by the different approaches for all animals studied at 24 h (n = 32) can be seen in supplementary figure S4. Here ANOVA found a significant difference between the segmentation approaches for the Training group (n = 12, ANOVA: F(3, 44) = 14.8, P < 0.001. Bonferroni correction shows significantly overestimated volumes by both ADC (P < 0.01) and T2W thresholding (P < 0.01). Likewise, ANOVA applied to the Testing group (n = 20) showed significant differences among the segmentations F(3, 76) = 21.1, P < 0.001, again with ADC and T2W thresholding overestimated the stroke volumes (P < 0.01) while ML and Manual ROIs showed no significant differences.

### Histological findings and correlations to stroke volume predictions

**Fig 2:**
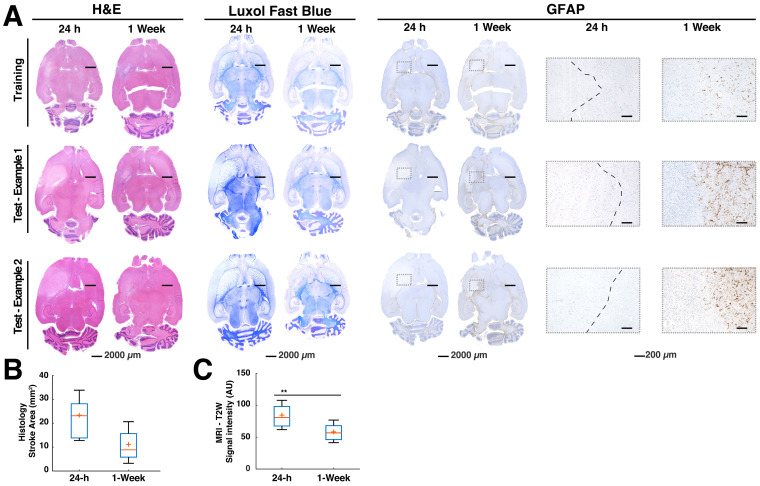
** Histology examples and quantification.** The histology and immunohistochemistry data demonstrate findings of pathology performed on the animals at 24 h and 1-week time points post stroke. **(A).** The H&E showed predominantly large edematous areas at 24 h in all groups. This could also be observed in the luxol fast blue staining, which allows the differentiation of white matter tracts in the brain and eases the visualization of structural changes. Glial fibrillary acidic protein (GFAP), a marker for determination of glial cell activation could not be observed at the 24 h time point, denoting a lack of neuroinflammation. The 1-week evaluations using H&E confirmed a reduction of the edema in all groups. Luxol blue further corroborated these reductions and showed a smaller infarct core in comparison to 24 h. GFAP showed activated glial cells confirming neuroinflammation around the ischemic core (boxes). The boxes specifically shown for GFAP demonstrate the lack of glial cell activation at the early stroke time point. A capsule of activated glia could often be observed in most of the regions surrounding the stroke core 1 week after stroke **(B).** A quantification of the stroke areas drawn at 24 h and 1-week time points by a pathologist using H&E estimated 50% reduction of the median stroke area (n=9 per group). ANOVA did not find significant differences between both time points. **(C).** Signal intensity of T2-weighted (T2W) images of the stroke regions belonging to the mismatch between the Manual region of interest (ROI) at 24 h and the histological ground truth found at 1-week post stroke (n=9 per group). There was a significant signal intensity reduction (P=0.04), representative of reduced water content in the regions consistent with subduing edema. The boxplots present several measures in symbols: Mean (+), median (red line), 1^st^ quartile of the data (box upper border), 3^rd^ quartile of the data (box lower border) and 15% outmost border of the dataset (whisker).

Histology confirmed stroke lesions only on the ipsilateral hemisphere of the MCAO in all the brains, consistent with the ML predictions. A more detailed description of the histological findings can be found in supplementary material. Most importantly, there were significantly large edematous stroke areas at 24 h in contrast to the samples acquired at 1-week post-stroke (Fig. 2A and 2B). Moreover at 1-week, glial cell reactivity increased surrounding the stroke area in most brains (Fig. 2A, rightmost panel). These findings were consistent with the significantly increased T2W signal intensity at 24 h in comparison to the 1-week in MRI (p < 0.01, Fig. 2C). The congruency between histology and T2W-MRI led us to examine the correlation between the Manual ROI volumes at 24 h and the 24 h histology areas defined by pathologists. We also examined the correlation between ML stroke volumes and 24 h pathology ROIs (subset of animals sacrificed at 24 h, n = 9). Very interestingly, Manual ROIs at 24 h and the ML predictions showed Pearson corresponding correlations of 0.70 (P < 0.05) and 0.85 (P < 0.01) to the areas calculated in 24 h histology. In stark contrast, thresholding of ADC and T2W images at 24 h showed lower Pearson correlations of 0.44 (ns) and 0.55 (ns), respectively. Altogether, histology confirmed the presence of stroke regions on the ipsilateral hemisphere to the occlusion and the ML segmentation volumes strongly correlated to histology at 24 h in contrast to automatized and manual segmentations.

### Final stroke volume prediction and comparisons to 1-week EGT

**Fig 3:**
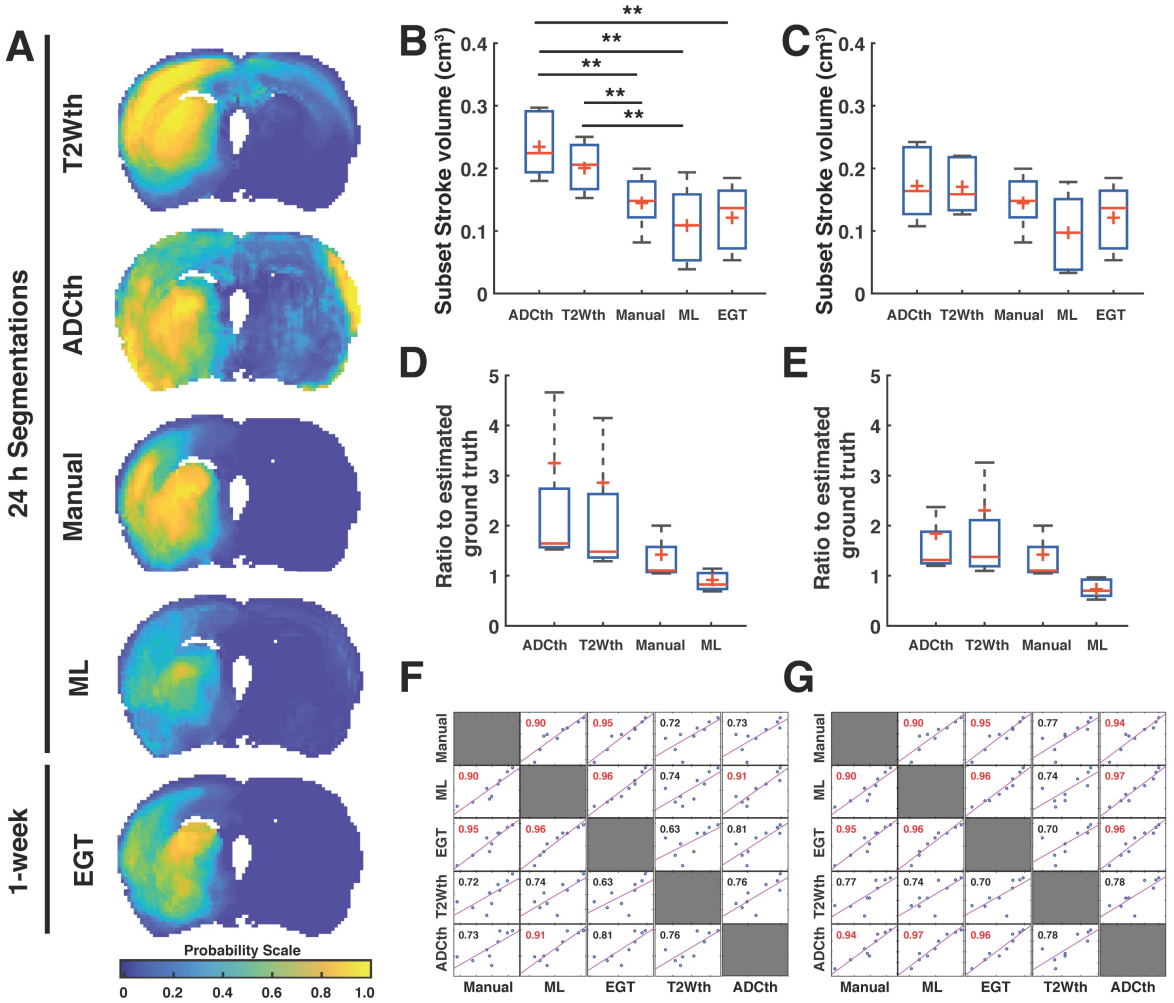
**Stroke volume comparisons for subset of animals validated with ground truth.** The data from the 24 h volume predictions belonging only to the subset of animals studied with MRI after 1 week is shown in the figure **(A).** The joint probability map of all the animals per group (n=9) are shown for all segmentation methods. This visualitation shows the consistency of stroke location on the territory of the middle cerebral artery. In this way, it is clear that apparent diffusion coefficient thresholding (ADCth) and T2-weighted image thresholding (T2Wth) largely overestimate stroke and misclasify areas in the contralateral hemisphere to the artery occlusion in comparison to the machine learning (ML) predictions, Manual segmentation (Manual) and estimated ground truth (EGT). **(B).** The boxplot graph the voxels classified as stroke on both hemispheres using the different segmentation approaches. ANOVA found significant differences between the groups (P<0.01) with both thresholding methods being significantly larger than ML and EGT **(B).** Shows the same data as the previous boxplot excluding the voxels from the contralateral hemisphere, further emphasizing the benefit obtained by the thresholding approaches by removal of the affected hemisphere. Significant differences were found in ANOVA (P=0.03), though bonferroni correction found no specific differences between the groups. **(C).** The boxplots show the ratio of the volumes to the EGT. In this case, no significant differences between the groups were found. **(D).** Evaluation of the ratio to the EGT as in panel C, without the contralateral hemisphere showed similarly no significant differences. **(E).** Correlation matrix of the stroke volume predictions using the different segmentation approaches using data from both brain hemispheres. Every data point represents the stroke volume of one animal. Most important of all is the comparison of the different methods to the EGT. Manual and ML segmentations were found to have the strongest correlations to EGT. ADCth and ML correlate strongly, which denotes the importance of ADC in the ML framework. Also noteworthy is that T2Wth and ADCth do not independently present a stronger correlation to the EGT than Manual or ML. **(F).** This correlation matrix is analogus to panel E except that contralateral hemisphere voxels are excluded. The ML volumes change only slightly and the correlation to EGT remains the same. The Manual approach does not change, since the experimenter segmented only the ipsilateral stroke hemisphere. T2Wth profits from the removal of the contralateral hemisphere, while ADCth dramatically increases its correlation to EGT. Pearson's correlations shown in red symbolize a P-value < 0.0001. The boxplots present several metrics in symbols: Mean (+), median (red line), 1^st^ quartile of the data (box upper border), 3^rd^ quartile of the data (box lower border) and 15% outmost border of the dataset (whisker). *P<0.05, **P<0.01.

The joint probability maps show the superposition of all animal probability maps as a common probability map in anatomical space. We focused now only on the group of animals that received a follow-up MRI at 1 week (Subset with n = 9) in order to compare all the segmentation maps to the EGT. The joint probability maps corresponding to each segmentation approach can be observed in Fig. 3A. Here, the stroke lesions can be seen in the left hemisphere, corresponding to the spatial distribution of the targeted middle cerebral artery territory. The findings are consistent with the analysis involving all animals with scans at 24 h (n = 32, supplementary Fig. S4), T2W thresholding (T2Wth) and ADC thresholding (ADCth) clearly overestimate the stroke regions on the contralateral hemisphere as compared to the Manual ROI at 24h, ML and EGT. Here, ANOVA showed a significant difference between the groups F(4, 40) = 8.23, P < 0.001. Multiple comparison post-hoc analysis revealed that thresholding approaches overestimated stroke volumes in contrast to EGT, Manual, and ML segmentations (Fig. 3B). The stroke volume ML predictions were not significantly different to the EGT volumes (MRI at 1 week) or the Manual ROIs at 24 h (Fig. 3B).

We quantified the voxels belonging only to the ipsilateral hemisphere to the occlusion (Fig. 3C). This greatly benefited the thresholding approaches while ANOVA still found significant differences between the methods (F(4, 40) = 1, P = 0.03). However, Bonferroni correction revealed no specific group differences. We plotted the ratio of the stroke volumes produced by every method to the EGT volume with and without the contralateral hemisphere (Fig. 3D-E). This ratio allowed a better visualization of the overestimation produced by thresholding; however, ANOVA found no significant difference between the groups. The data shows a slightly elevated ratio for the Manual ROI and a median ratio below one for the ML prediction. Altogether, these analyses show that the proposed ML approach predicts less false positive voxels on the non-stroke hemisphere than the thresholding approaches.

**Fig 4:**
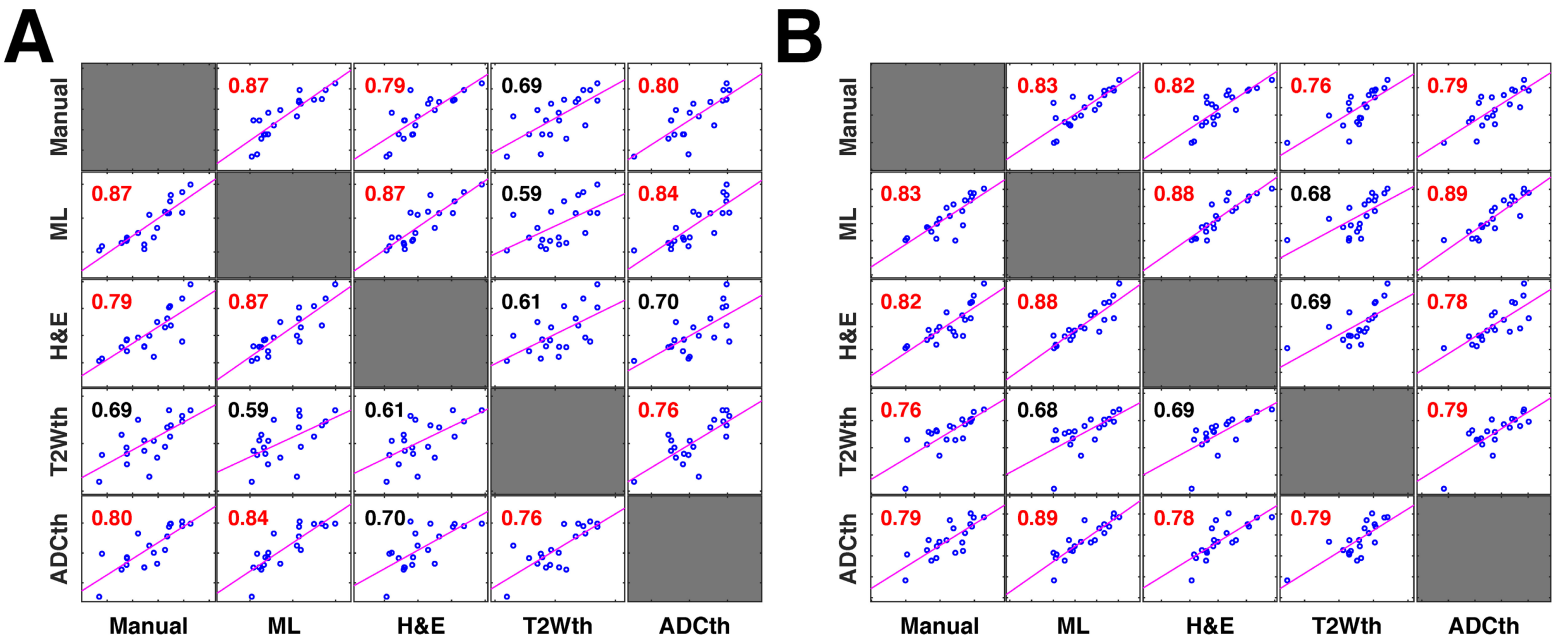
**Correlation plots of all stroke volume quantification methods to 1-week histology.** The correlation matrices show the relationship between the stroke volume measured by the different segmentation approaches in MRI and their relationship to the area directly measured by pathologists in 1-week histology (n=20 per comparison)** (A).** In this comparison the entire stroke volume measured in MRI was compared to the single histology area. Both the machine learning (ML) and Manual ROI stroke volumes have a strong correlation to the corresponding H&E areas. In comparison, thresholding approaches using apparent diffusion coefficient (ADCth) and T2 weighted images (T2Wth) show poor correlations. These correlation relationships are similar to the ones in Fig. 3E and 3F, however, this analysis contains a larger data sample. Here, ADCth once again shows a strong correlation to the ML predictions; also ADCth and T2Wth by themselves do not correlate strongly to H&E. **(B).** In this comparison only one slice of the MRI Volume per animal with corresponding H&E slice (or ground truth) was used to calculate the correlations. This approach provides the best comparison between the methodologies. Here, it can be observed that ADCth correlates strongly to the ML prediction and to the H&E. Pearson's correlations shown in red color have a p-value < 0.0001.

**Fig 5:**
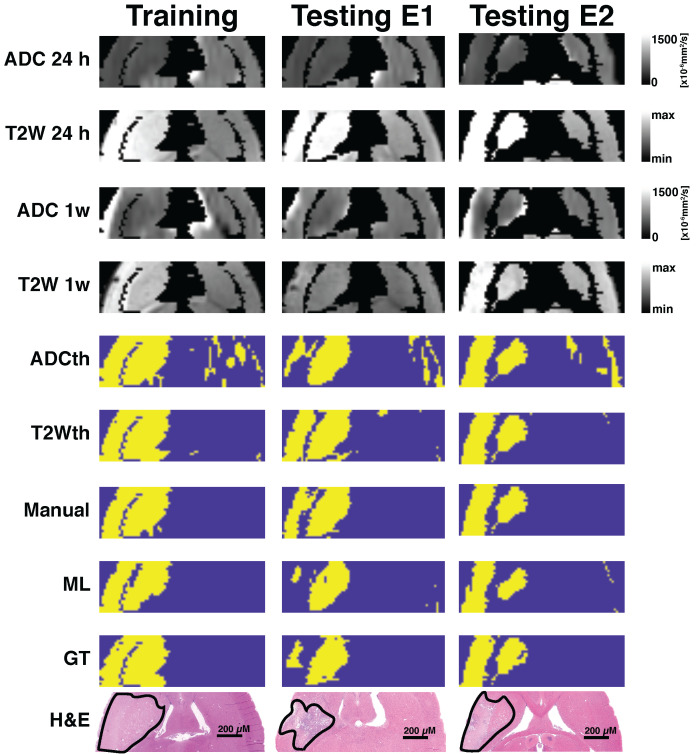
**Exemplary images of animals belonging to the different cohorts.** One training animal and two testing animal examples are shown side by side. These animals were scanned with MRI at 24 h and 1-week post-stroke followed by histological evaluations. Here the apparent diffusion coeffcient (ADC) and T2-weighted images (T2W) are shown for both time points. Thresholding predictions are shown as ADCth and T2Wth. The manually drawn region of interest (Manual) using 24 h data demonstrates that the combined analysis of both ADC and T2W images at 24 h predicts a very similar to histology and ground truth (GT). The machine learning (ML) prediction has a very similar shape as that of the Manual ROI and the GT. Overall, the thresholding approaches tend to overestimate the final stroke volume at 1-week post stroke, while the Manual ROI and ML predictions closely resemble the final histological outcome and GT.

Next, we investigated 1-week follow-up subset group (n = 9) by calculating the volume correlations between the total stroke volumes segmented at 24 h and the 1-week whole brain stroke volume of the EGT (Fig. 3F). Both the ML and Manual ROI presented the strongest correlations to EGT volumes of all segmentations. Again, removal of the contralateral hemisphere increased the correlation of the thresholding approaches (Fig. 3G). In order to further validate these volume relationships, we correlated the whole-brain stroke volume segmentations at 24h with the mm^2^ area directly delineated by the pathologist on 1-week H&E slices, which also counted with in a larger data sample (n = 20, Fig. 4A). Consistent with the subset data (n = 9), the strongest significant correlation was found between EGT and ML predictions (Pearson = 0.87, P < 0.0001) followed by the Manual ROI at 24 h timepoint (Pearson = 0.79, P < 0.0001). Finally, to provide a stricter one-to-one validation of MRI and histology (ground truth), we correlated the H&E stroke area at 1-week (n = 20) with the matching MRI-slice at 24 h (Fig. 4B). The areas calculated by the pathologist in H&E correlated best to stroke region found in the matching MRI slice of ML (Pearson = 0.88, P < 0.0001) followed by the Manual segmentation (Pearson = 0.82, P < 0.0001). This relationship can be best visualized in the detailed comparisons of the MR images in Fig. 5, which shows the different segmentation approaches and the corresponding H&E slices. In summary, the ML approach, independent of the prior knowledge of the stroke hemisphere location segmented similar stroke volumes to manual segmentation and which strongly correlated with histological outcomes at 24 h and at 1-week post stroke. In stark contrast, threshold-based segmentations overestimated stroke volumes and presented poor correlations to histology.

### Metrics of prediction success

In order to further evaluate which of the segmentation methods best classify stroke from non-stroke tissue, we performed voxel-wise evaluations of the Dice Similarity Coefficient (DSC), accuracy, Matthew's Correlation Coefficient (MCC), sensitivity, Positive Predictive Value (PPV), False Discovery Rate (FDR), Negative Predictive Value (NPV), specificity and False Positive Rate (FPR) of all the methodologies.

First, we calculated the success metrics between the ML predictions and the Manual ROIs produced through MRI and histology at the 24 h timepoint (n = 9). These evaluations produced a median DSC of 0.32, accuracy of 0.83, MCC of 0.33, sensitivity of 0.20, PPV of 0.70, FDR of 0.28, NPV of 0.84, specificity of 0.98, and FPR of 0.01. We suspected that metrics were affected by the significant edema (Fig. 2A-C), especially DSC. Moreover, since final stroke volume has been quantified until 1-week after stroke onset 19, we turned our focus to the metrics evaluation using 1-week histology.

**Fig 6:**
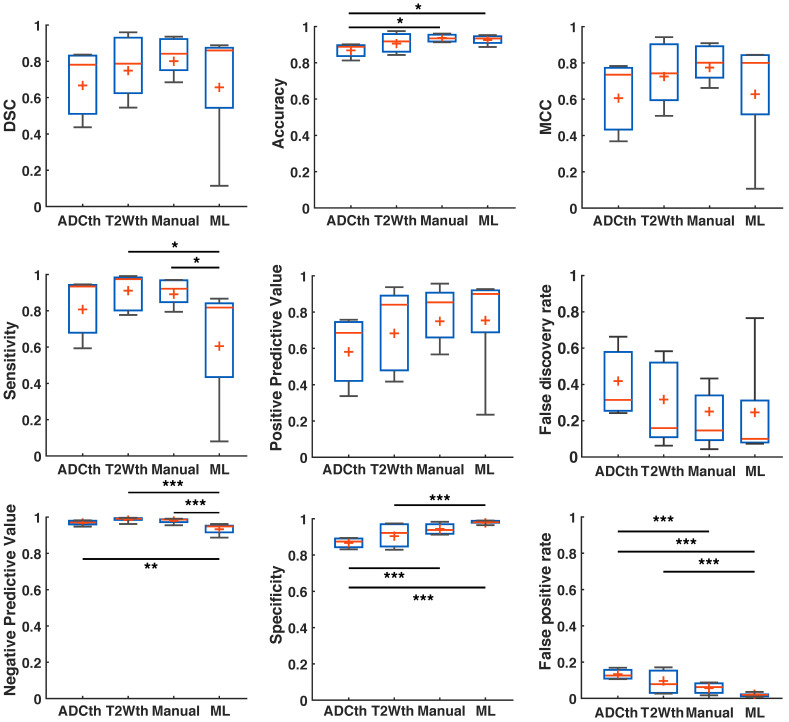
**Metrics detailing stroke volume (SV) prediction in preclinical data.** The boxplots present several measures in symbols: Mean (+), median (red line), 1^st^ quartile of the data (box upper border), 3^rd^ quartile of the data (box lower border) and 15% outmost border of the dataset (whisker). Dice similarity coefficient (DSC), Mathew's correlation coefficient (MCC), positive predictive value, false discovery rate showed no significances in ANOVA. The median values, however, are slightly increased in the ML group in comparison to that of the other groups, except in false discovery rate where it the median is the lowest. ANOVA showed significant differences in the metrics of accuracy, sensitivity, negative predictive value, specificity and false positive rate. Here, for the most part ML and Manual segmentation significantly outperformed thresholding methods. Sensitivity is significantly reduced for the ML group in comparison to the thresholding methods. *P<0.05, **P<0.01.

There was a remarkable improvement in the metrics between the different segmentations and EGT (Fig. 6). ANOVA found significant group differences in Accuracy (F(3,32) = 4.27, P = 0.01), Sensitivity (F(3,32) = 3.91, P = 0.02), NPV (F(3,32) = 8.9, P < 0.001), Specificity (F(3,32) = 11.2, P < 0.001) and FPR (F(3,32) = 11.2, P < 0.001). Multiple comparison significances and confidence intervals are detailed Fig. 6 and supplementary Table S2. ML presented a significantly lower FPR and NPV, as well as the highest specificity of all approaches. Median DSC, Accuracy, MCC, PPV, and FDR were similar between ML and Manual ROI approaches. Median DSC, PPV, MCC and Accuracy were higher in the ML prediction as compared to thresholding approaches. Here, however, ANOVA found no significant differences. Altogether, the metrics showed that ML outperformed the thresholding approaches in regards to differentiation of false positives and for the most part behaved similarly to Manual segmentations.

### Clinical Translation: Stroke identification on human data

**Fig 7:**
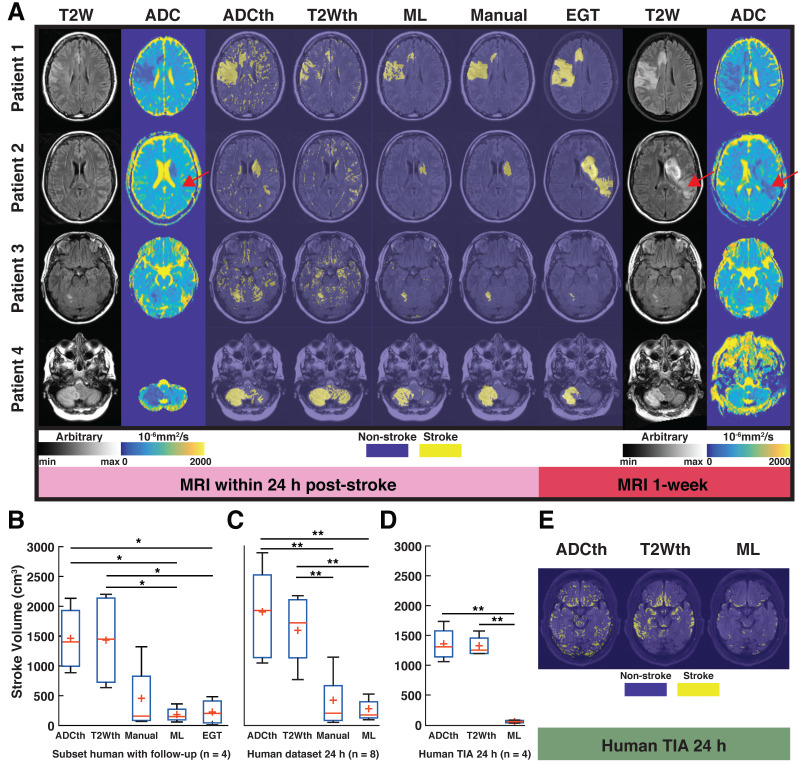
**Human translation examples.** All the different segmentations approaches and stroke volume quantifications are shown. **(A).** The main figure shows images the four patients with ischemic stroke with MRI follow-up at 1-week post stroke. The left panel shows the 24 h MRI and the different segmentations performed while the right panel shows the estimated ground truth (EGT) and follow up MRI. T2-weighted (T2W) images and apparent diffusion coefficient (ADC) maps are shown with slices corresponding to ADC and T2 thresholding (ADCth and T2Wth), machine learning (ML) and Manual segmentations. The first example shows a stroke delimited to the right hemisphere in ADC and T2W images. For the most part there is good correspondance between ML, Manual segmentation and EGT. Except, for the second patient which presented a novel stroke region sometime after the scan (red arrow). **(B).** ANOVA showed that the quantification of the stroke volume of the subset of patients was significantly different between the segmentation approaches (P<0.01), especifically the thresholding approaches overestimated the stroke volumes in comparsion to ML and EGT. **(C).** The evaluation of the 24 h stroke volume quantification using all studied stroke patients (n=8) also yielded significant differences using ANOVA (P<0.001). Here, Manual segmentation was also significantly different to the thresholding approaches. **(B).** Finally, ANOVA (P<0.001) showed that the evaluation of patients with transient ischemic attack (TIA - no stroke region in MRI), presented significantly more false positive stroke regions using thresholding approaches than ML. *P<0.05, **P<0.01.

MRI and the results of different segmentation methods for the four stroke patients that received follow-up scans can be observed in Fig. 7A. The left panel in Fig. 7A shows the 24 h clinical datasets (first two columns) and the right panel (last two columns) shows the follow-up images recorded approximately 1-week after stroke. Patient 1 shows decreased ADC in the stroke lesion, whereas the T2W image shows slightly increased signal intensity, consistent with acute IS. Here, the stroke regions predicted by ML and the radiologist at 24 h are very similar, whereas T2Wth and ADCth overestimate the stroke regions. The 1-week follow-up images show that the stroke region presented a slight increment in extent and an increased signal intensity in the T2W image. Moreover, diffusion restrictions are still present after 1-week. The second patient shows that ML predicted a stroke region with a similar shape and size as the radiologist at 24 h. Seemingly, the clinicians weighted the decision of stroke delimitation based on the ADC pattern, disregarding the lack of T2W signal increment. In this case, the algorithm also more closely resembles ADC reductions and disregards the lack of T2W signal increment, as in the first patient. Importantly, the second case shows a remarkable increased stroke extent in the follow-up scan. The novel stroke extent at 1-week was diagnosed as a new onset of acute stroke. In this case, T2th and ADCth misclassify many voxels due to the imaging artifacts. The third patient shows a small, focal lesion in the cerebellum, which slightly changes at 1-week. The identification in the cerebellum is important, since it shows that the ML can identify strokes in other brain regions. Finally, patient 4 also shows a lesion in the cerebellum with decreased ADC and increased T2W values at 24 h and a normalized ADC and increased T2W signal intensity at 1-week post stroke.

ANOVA of the subset of patients with follow-up, found significant differences among the stroke volumes calculated by the segmentation methods F(4,15) = 5.39, P < 0.01). Again, ML and EGT presented stroke volumes similarly smaller than ADCth (P < 0.05) and T2Wth (P < 0.05) as seen in Fig. 7B. The analysis of the complete 24 h dataset (Fig. 7C, n = 8) was consistent with these findings (F(3,28) = 5.39, P < 0.001), where the different relationships between thresholding, manual and ML were maintained. Focusing on patients with TIA (no MRI lesion), we measured the amount of scattered single stroke voxels segmented by the automatized methods (Fig. 7D-E). ANOVA found that ML presented significantly less false positives than the thresholding approaches F(2,9) = 49.7, P < 0.001).Taken altogether, the proposed ML algorithm successfully identified stroke volumes in the eight patients with radiologically confirmed acute lesions and did not identify focal stroke lesions in the four patients with suspected TIA.

**Fig 8:**
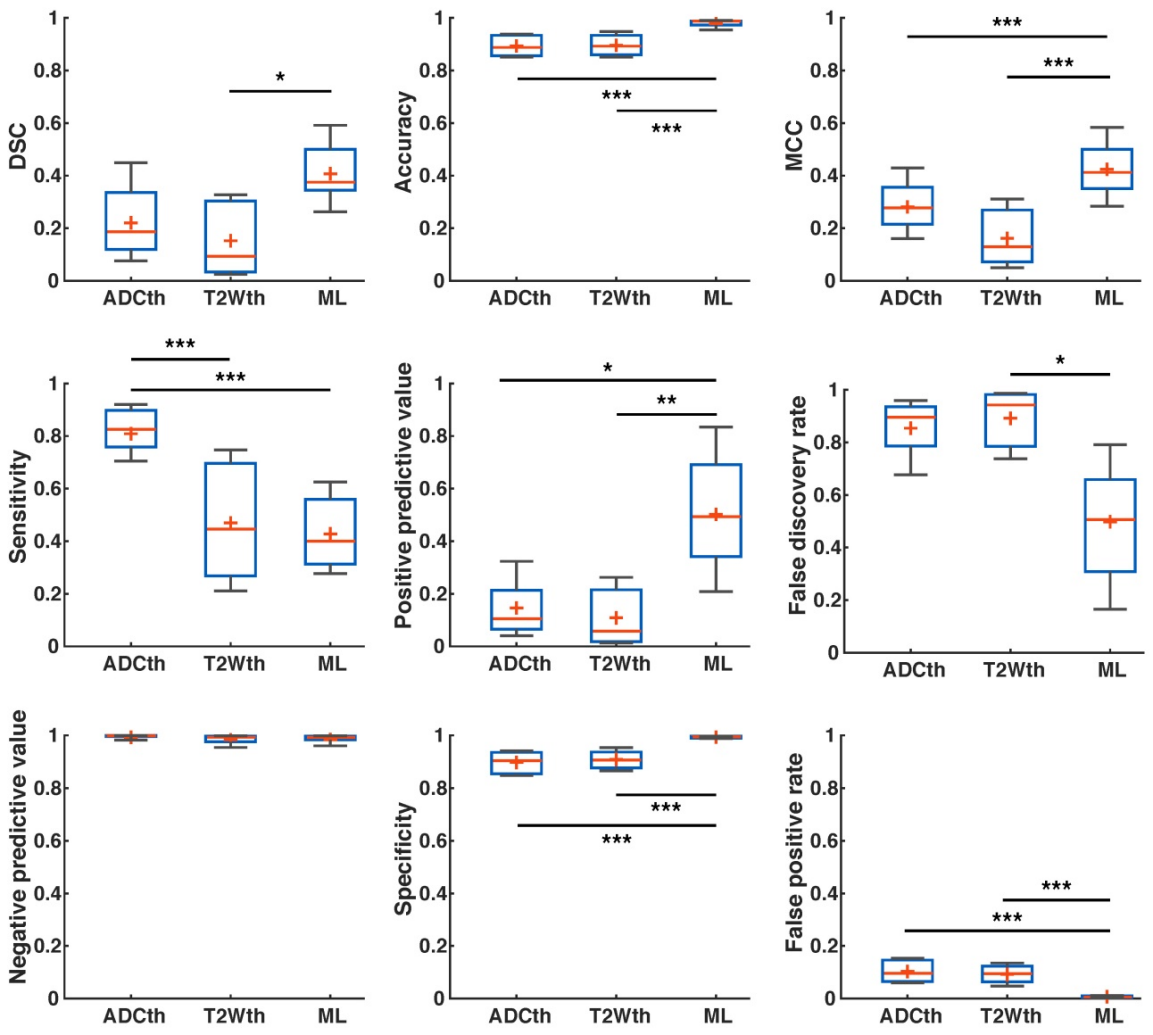
** Joint probability maps.** The overlay of the strokes of every animal per group are shown as a probability of stroke occurrence for ease of visualization as depicted in the probability scale. Here, it is clear that apparent diffusion coefficient thresholding (ADCth) and T2-weighted image thresholding (T2Wth) largely overestimate stroke and misclasify areas in the contralateral hemisphere in comparison to the machine learning (ML) predictions. At the same time the consistency of stroke location on the territory of the middle cerebral artery can be observed.

**Fig 9:**
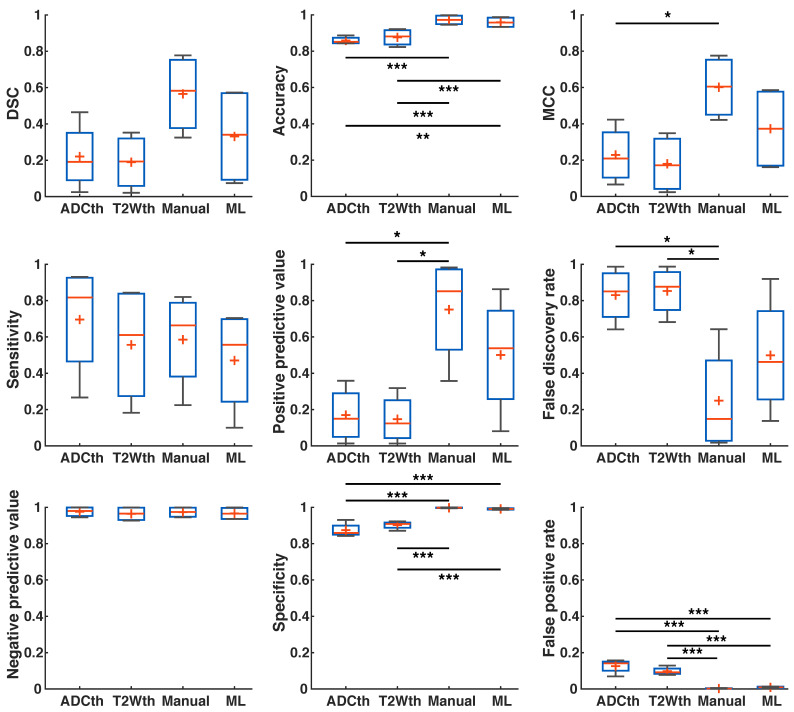
**Metrics of prediction success in humans at 1-week.** ANOVA found a significant difference in Accuracy, specificity and false positive rate between the groups, with Manual segmentation and ML outperforming thresholding. Manual segmentation outperformed all approaches as measured by Matthew's correlation coefficient (MCC), positive predictive value and false discovery rate. Here, ML performed better than thresholding approaches but not as Manual segmentation. ANOVA showed no differences between the groups in Dice similarity coefficient (DSC), sensitivity and negative predictive value. Manual segmentation presented the highest median DSC of all approaches, while ADC thresholding (ADCth) presented the highest sensitivity. Median negative predictive values were the similar for all approaches. The boxplots present several measures in symbols: Mean (+), median (red line), 1^st^ quartile of the data (box upper border), 3^rd^ quartile of the data (box lower border) and 15% outmost border of the dataset (whisker). *P<0.05, **P<0.01.

Using the human stroke-brain datasets, we calculated the metrics of prediction success of all segmentation methods against Manual ROI segmentations produced with 6-24h MRI (n = 8) and 1-week follow-ups (n = 4). The 24 h metrics and statistical differences can be seen in Fig. 8 and supplementary Table S3 and S4. One-way ANOVA showed significant differences (P < 0.05) for all metrics at 24 h except NPV. Bonferroni corrected comparisons showed that ML presented significantly higher accuracy (P < 0.001), MCC (P < 0.001), PPV (P < 0.001) and specificity (P < 0.001) than both thresholding methods. FDR and FPR were also significantly lower for ML than for both thresholding methods. As performed in animals, we focused on the final stroke volume at 1-week using EGT. These results are shown in Fig. 9 and confidence intervals are shown in supplementary Table S5 and S6. Here, ANOVA showed significant differences (P < 0.05) for all metrics except DSC, Sensitivity and NPV. Bonferroni corrected comparisons showed that ML achieved a higher accuracy and specificity (P < 0.001) than both thresholding approaches. Median MCC, PPV and DSC were also higher than the thresholding approaches, but showed no significant differences in multiple comparison. Manual delineation at 24 h still presented higher DSC, Accuracy, MCC, PPV and Specificity than all other approaches. Altogether, both the 24 h and 1-week time points show that the ML algorithm presented better segmentation performance than the thresholding approaches and similar performance to Manual segmentation.

We noticed that the ML framework made few but considerable false predictions on the contralateral brain hemisphere and on the suspected TIA patient data. This false positive “noise” further affects the metrics of success, especially DSC, which measures shape similarity. In supplementary data we show that by applying a simple median filter as post-processing step, this voxel-level noise can be significantly reduced, leading to improvements in the metrics of success.

## Discussion

Brain lesion image segmentation is essential in multiple fields of neurology. Quantification of stroke volume plays a central role in clinical and preclinical drug trials and neuroprotection, while accurate localisation plays a role in neuroplasticity, neuroimaging biomarker research, neuroinflammation, rehabilitation and neuromodulation research. We propose here an easily reproducible and robust methodology that can directly benefit clinical and preclinical stroke research in these fields.

Using a simple clustering approach, we successfully identified the known ADC and T2W signal intensities corresponding to IS lesions 24 h after stroke induction 17,42. The stroke cluster identified by GMM was conservative and contained prominent and relevant ADC and T2W characteristics, making the algorithm simple and robust. Our data shows that stroke volumes predicted in rats by ML were slightly smaller, but not significantly different to manual delineations at 24 h or EGT. On the contrary, thresholding approaches showed overwhelmingly larger stroke volumes. The thresholding approaches widely and falsely classified a significant number of voxels as stroke on the contralateral hemisphere (Fig 3A). In rats, when using 24 h segmentation as ground truth, the metrics are not so favourable for ML as reflected by the reduced median DSC of 0.32. We hypothesize that this is due to broad regions of edema at 24 h, as compared to the 1-week timepoint (Fig. 2). It appears that the ML segmentations disregard edema at 24 h leading to stronger correlations and similarities in comparison to GT, than in comparison to manual delineations at 24 h (Fig. 3-5).

Our findings provide strong evidence that single-parameter approaches do not reliably segment pathological tissue changes (Fig. 3-6). One of the reasons for their sub-optimal performance is that a single parameter does not functionally or spatially cover all the necessary target-tissue characteristics and over- or underestimates others. The joint probability maps and boxplots in Fig. 3 show a large number of voxels misclassified as stroke by single-parameter approaches. Multiparametric approaches, such as GMM and RFC, can overcome these limitations because they are able to utilize complementary tissue characteristics captured by different imaging modalities. For example, the intersection between reduced ADC and T2W signal hyperintensity allows the identification of strokes in the rats in supplementary figure S3 and in the patients in Fig. 7. The ML algorithm discerns in preclinical data false positives quite robustly. This discernment is for example evident in the stroke lesion identified in the exemplary brain in supplementary figure S3 (Testing E2), which shows a reduced ADC artefact (red arrow) correctly classified as non-stroke due to normal T2W signal intensity. In the same vein, the algorithm correctly classifies a stroke lesion in the striatum of Patient 2 at 24 h (Fig. 7) without T2W signal hyperintensity. These examples portray the range of tissue similarities encompassed by the algorithm to perform the predictions, which are far more robust than commonly used thresholding approaches.

In the clinical datasets, ML stroke volume was consistent with manual segmentations and EGT. The thresholding approaches widely overestimated stroke volume in contrast to ML, in patients with stroke and with suspicion of TIA (Fig. 7). Moreover, ML outperformed the thresholding approaches in almost all success metrics at 24 h. Most notably and consistent with the preclinical data, the framework presented significantly lower FPR and significantly higher PPV, specificity and accuracy than the thresholding methods. Unexpectedly, we found that the clinical metrics of the ML algorithm were better at 24 h than at 1-week in contrast to the animal datasets. This could have several explanations, which highlight certain limitations of our study. The first and most apparent explanation is that edema plays a larger role in animal models due to the smaller brain size, in contrast to the disproportionate lesion size in humans (in our data). Another explanation is the different number of patients (n = 8) at 24 h and at 1-week (n = 4) timepoints plays a statistical role. Unfortunately, 1-week MRI follow-ups are scarce, since there are no clinical indications for biopsy and necessary prospective studies. Moreover, in the clinic, the time of stroke onset is not certain (6 - 24 h). This uncertainty entails that the relationship between ADC and T2, for which the ML framework is trained in animals, is not optimal, and therefore the stroke volume prediction evidenced in rats may be lost. Moreover, there is no histological validation for the manually produced ROIs in humans at any time point, thus the ground truth itself is not validated. Moreover, due to the lack of histology, for humans the whole stroke volume was used for metric calculation, while in the animal, it was specifically performed on the MRI slide with histological validation. Besides these factors, another important aspect to note is that biologically, human lesions are far more complex than the MCAO model. Ideally, the stroke model complexity should match pathophysiological complexity in order to better simulate the disease. For the most part, the lesion evolution of the patients with follow-up was somehow delayed (T2W hyperintensity maximum at 1-week) in comparison to the lesion evolution of the MCAO rat mode 13,42. An explanation is the lack of “true certainty” of the time of stroke onset, which sets the starting point for the tissue swelling pattern. Moreover, the site of occlusion, ongoing treatment, haemorrhagic transformations and other clinical complications are all important factors. For example, Patient 2 presented an additional stroke lesion sometime between 24 h and the 1-week scan. In stark contrast, the MCAO rat model is not designed to evolve secondary stroke lesions and the lesion severity is homogenous due to a set occlusion time in similar age healthy rats. Therefore, the clinical case highlights both, the difficulty in predicting final stroke volumes in humans and the challenge to accurately model stroke in animals. One way to improve human lesion identification using this framework, could be to train stroke data with variable lesion evolutions and various grades of lesion severity in animals to produce more heterogeneous datasets for testing in humans.

In this study, we determine multiparametric MRI features of acute stroke in an unsupervised manner by applying a GMM on the imaging data of control rats. These results were subsequently used to train an RFC and detect stroke regions in independent preclinical and clinical testing cohorts. The performance of the trained RF classifier is therefore limited by the accuracy of the GMM clustering results obtained in the first step. Furthermore, as an alternative, one could also apply the identified GMM directly to the testing cohorts. However, our approach of training a separate RFC for testing is motivated by two main reasons. First, training such a classifier allows to easily combine labelled datasets from different sources, which would not be possible using the mixture model. For example, in an active learning framework, it is desirable to combine human delineated annotations (preferably on difficult examples from the training set) with labels obtained from unsupervised learning algorithms, and train a single machine learning model while maintaining the performance on the test set 62. In the same vein, our approach can be used to augment training sets in a clinical setting by adding pre-processed data from matching preclinical trials. As we here show the translational feasibility of the preclinical stroke features, this direction can be of further interest in the neurology field. Secondly, the RFC is algorithmically suitable for parallel computing, which makes it scalable and computationally efficient for large clinical cohorts. Besides the GMM-derived labels, one can also use the GT to train the RFC, however, in our study this would have limited the training dataset to a single imaging plane per animal brain (for which the histology was available). Using the proposed workflow enables the usage of the entire brain volume.

Thresholding has limitations in the clinical scenario, not only due to the large overestimation of the stroke regions, but because its application may be limited, since stroke (e.g. due to cardiac embolism) might entail multiple focal locations on different brain hemispheres. In these cases, the multiparametric ML framework can be a more reliable tool for the clinician to exclude the presence of ischemic lesions. Moreover, fixed thresholds may oversimplify the wide ADC and T2W distributions found in different human populations with different ages and disease states. By reducing the examiner bias, our method can be used as an automated, time-effective, objective and robust tool for stroke quantification.

Few research groups have segmented stroke regions and validated their results with histology 45,46. Previously performed stroke volume segmentations have shown positive correlations to histological scoring (disregarding spatial metrics), but weaker correlation to histological stroke areas. 46. Moreover, these studies disregarded temporary spatial changes produced by edema evidence, since they did not aim at predicting final stroke volume 45,46. In humans, various groups have successfully implemented complex stroke segmentation approaches using different ML methods (lacking histological validation) by training multiple, often not-widely used clinical MRI parameters or by relying on large scale datasets 49,50,52-54. Our work particularly stands out from previous publications by providing preclinical longitudinal multiparametric MRI acquisitions and substantiating our segmentations with histological quantification. Rate of tissue degeneration during stroke is multifactorial, which is understandably why other groups have implemented segmentation approaches using numerous specialized MRI parameters. However, this can represent difficulty for widespread applicability and reproducibility by other institutions. In contrast, our approach effectively segments stroke at 24-h post stroke using two routinely applied MRI parameters, both in preclinical laboratories and in the clinic. We further demonstrate that our ML segmentations significantly overperform commonly performed thresholding segmentations. Moreover, in rats, the segmented stroke lesions at 24 h are compatible with 24 h manual segmentations and final stroke volume measurements at 1-week. Finally, our work is explicitly different, since we apply for the first time a new approach to validate clinical datasets using animal histology. Though modifications, algorithm improvements and larger sample testing need to be performed to optimize the algorithm for clinical work, our simple approach opens the door to train more complex setups and improve stroke segmentation in humans.

Our findings indicate for the first time that the microstructural and functional information stored in MRI data (as measured by ADC and T2W) can be directly translated between species. This method of translation using ML holds high importance for medical science because it reduces the gap between the clinical and preclinical fields by allowing the analysis of human studies and animal experiments side-by-side. This novel approach proposes to circumvent the lack of histological validation in human imaging datasets with a novel non-invasive alternative. At the same time, such a framework might enable a more accurate design of clinical follow-up trials, as well as design of experiments that utilize animal data as ground truth. Therefore, the proposed method can help in improving the fundamental understanding of the differences between animal models and the actual human disease.

## Supplementary Material

Supplementary methods, data, figures and tables.

## Abbreviations

ADC: apparent diffusion coefficient
ADCth: adc threshold segmentation
ANOVA: analysis of variance
DSC: Dice similarity coefficient or index
EGT: estimated ground truth
FDR: false discovery rate
FPR: false positive rate
GMM: gaussian mixture model
GT: ground truth
H&E: hematoxylin and eosin
IS: ischemic stroke
MCAO: middle cerebral artery occlusion
MCC: matthew's correlation coefficient
ML: machine learning
MRI: magnetic resonance imaging
NPV: negative predictive value
PPV: positive predictive value
RFC: random forest classifier
ROI: regions of interest
T2W: t2-weighted
T2wth: t2w threshold segmentation
TTC: triphenyltetrazolium chloride
