# Supplementary material for: Machine learning identifies stroke features between species
Source: Theranostics. 2021 Jan 1;11(6):3017–34. doi: 10.7150/thno.51887 (PMC7806470; doi:10.7150/thno.51887)
Supplement: Supplementary file 1 — Supplementary methods, data, figures and tables. [file thnov11p3017s1.pdf]

Supplementary Materials

Animal handling, number and data distribution

A total of 34 male rats weighting between 322 and 376 g were used for the experiments. Day/light cycles were set to 12 h and food and water was provided *ad libitum*. Body weight measurements were followed daily following the surgical procedure. Animals were kept in groups of 4 animals in type IV Makrelen individually ventilated cages (IVC, Tecniplast, Hohenpeißenberg, Germany) with nesting material, wood chips and wooden enrichment according to German federal regulations. Animals had *ad libitum* access to standard pellet rodent food (Ssniff, Soest, Germany) and tap water. Cages were kept in a minimally disturbed room at 22 °C with 50% humidity following a light exposure of 12 h (day/night).

From the planned animals only 32 were evaluated using the machine learning framework. Two animals from the Training group were excluded due image related artifacts rendering them unusable for analysis. Furthermore, animal mortality claimed three histological samples on the Training (n=2) and Test group (n=1). The original neuroprotective trial was focused on immunohistochemistry and histology rather than MRI for the 1-week timepoint evaluation, therefore only 9 animals were scanned at 1-week using MRI. The rest of the animals used in the experiment are tabulated on Supplementary table 1.

Peri-Surgical Care

For surgery, animals were placed on an automatic temperature-controlled surgical surface (TC-1000 Temperature Controller, CWE Inc., USA) which maintained the animals at 37.0 ± 0.5 ˚C as monitored through a rectal probe. Isoflurane anesthesia at 5% induction and 1.5-2.5% maintenance was administered using a face-mask. Training and Test-group1 animals received isoflurane vaporized in a mixture of 30% oxygen and 70% room air at a flow rate of 1.5 L/min, whereas the Test-group2 animals received isoflurane using 100% O_2. The flow rate was maintained throughout the surgeries. During the surgery, blood pressure was monitored using a PE50 catheter which was inserted into the femoral artery. Furthermore, a small incision and thinning of the right parietal bone was performed, 2 mm posterior and 6 mm lateral from the Bregma suture using a surgical drill. This served in order to place a small probe to monitor cerebral perfusion using Laser Doppler (PeriFlux System 5000, Perimed Instruments, Sweden) throughout the surgery. Acute pain management for the surgical interventions was performed using s.c. administration of Buprenorphin 0.2 mg/kg 5 minutes before waking and 12 h after surgery.

Middle cerebral artery occlusion model – Stroke Induction

A middle cerebral occlusion with reperfusion, similar to the description by Longa et al. was used to induce ischemic stroke on the rats [1]. Briefly, surgeries were performed under a surgical microscope. The surgical neck area was shaved and disinfected, prior to the surgical incision. Xylocaine (1%) was injected locally, followed by a mid-line incision on the neck. The external carotid artery was dissected and surgical clamps were used to occlude proximal to the common carotid artery bifurcation and on the external carotid artery, distal to the common carotid artery bifurcation. A temporary ligation of the external carotid artery near the base of the bifurcation was placed to avoid retrograde bleeding. An incision was then made on the external carotid artery for the insertion of a silicon-coated nylon filament 0.33 ± 0.02 mm thick (Doccol Corporation, USA), which was pushed into the common carotid artery bifurcation and then into the internal carotid artery. Simultaneously, the temporary ligation was loosened to allow the passing of the coated filament into the skull towards the middle cerebral artery bifurcation; effectively occluding blood flow distal to it for 100 minutes. Laser Doppler flow was used to monitor reductions in cerebral blood flow before, during insertion of the filament

and during its removal. Following the occlusion period, the filament and clamps were removed allowing reperfusion and the surgical wound was closed.

Hypothermia Administration

The animals used in this manuscript originally were part of a neuroprotective trial consisting of whole-body hypothermia and oxygen administration. The trial yielded no significant differences between the groups. However, here we disclose the evaluations performed on them. The whole-body hypothermia treatment was administered directly after recanalization following MCAO surgery to the Testing-subgroup1, while the Testing-subgroup2 received whole body hypothermia treatment plus 100% oxygen administration.

Following surgery, animals in Testing-subgroup1and Testing-subgroup2 received systemic hypothermia therapy. This treatment was performed immediately after recanalization and consisted on the whole-body cooling of the rats to a temperature of approximately 32.6 ± 0.4 °C for 30 minutes. Using a Thermo/HAAKE C25P Refrigerated Bath system (Sigma-Aldrich, USA), cold water was pumped through a closed plastic-hose shaped as a cylindrical bore in which the rat was placed. The cooling system allowed the body temperature reductions which were closely monitored using a rectal probe (CWE Inc.). Training animals were maintained under the same conditions at 37.0 ± 0.5 °C.

Imaging Acquisitions – Preclinical

The animals were scanned 24 h after stroke induction using MRI. Anesthesia for the scans was performed using isoflurane (induction at 5% and maintenance at 1.5-2.5% vaporized in the room air at 1.5 L/min) and placed on an MRI-compatible bed with an integrated water heating system (Bruker Biospin, Ettlingen, Germany). Temperature was monitored using a rectal probe and maintained at 37.0 ± 0.5 °C. The heads were fixed on brain-dedicated MRI-beds using stereotactic pins. Respiratory rate was measured during the MRI acquisitions.

T2W images were acquired using a 3D-spoiled turbo spin echo sequence (161 × 256 matrix, 35 x 57 mm^2 field of view (FOV), repetition time (TR) = 3000 ms, echo time (TE) = 205 ms, slice thickness = 0.21 mm). Diffusion weighted images (DWI) for apparent diffusion coefficient (ADC) image calculations were acquired using an echo planar imaging (EPI) sequence on the coronal plane covering most of the brain (52 x 128 matrix, 21 x 54 mm^2 FOV, TR = 5500 ms, TE = 60 ms, Flip angle = 90°, Slice thickness = 1 mm, b-values = [0, 1000] s/mm^2 and 30 diffusion directions. As part of another evaluation, Perfusion measurements were also measured as previously described [2–4]. For this, one slice of coronal plane Flow-sensitive Alternating Inversion Recovery and a True Fast Imaging with Steady Precession (FAIR True-FISP) acquisition protocol (64 x 64 matrix, 25 x 25 mm^2 FOV, TR = 4.1 ms, TE = 2.05 ms, inversion time (TI) = 1800 ms, interscan time = 7000 ms, flip angle = 70°, number of inversions = 30, ST = 1.2 mm).

The rats also received an injection of 35 Mbq [^{18}F]FDG at 24 h as part of an additional experiment, where glucose metabolism in the stroke brain was evaluated. For injection and during the PET-scan, the animals were kept under Isoflurane vaporized in room air at 1.5 L/min and at a temperature of 37.0 ± 0.5 °C as previously described. After 1 hour of [^{18}F]FDG uptake under isoflurane anesthesia, the animals were scanned for 10 minutes on a dedicated small animal Inveon PET scanner (Siemens Healthineers, Knoxville TN, USA).

Imaging Acquisitions – Clinical

T2-weighted fluid attenuated inversion recovery (FLAIR) sequence was acquired using the following parameters: TR = 8800 ms, TE = 87 ms, TI = 2500 ms, ST = 4.5 mm. Diffusion

weighted images were acquired with an echo planar imaging protocol (TR = 5900 ms, TE = 93 ms, ST = 3 mm, 2 averages and 3 b-values= [0, 500, 1000] s/mm^2).

Preclinical imaging - Post-processing

The acquired diffusion weighted images were used to calculate ADC images using Syngo software (Siemens Healthineers, Erlangen, Germany). Signal intensity in T2W images was individually corrected by linearly fitting the decaying average image intensity and using the fit-slope for the correction to minimize the effects of signal loss in deep tissues as measured by the dedicated surface brain coil. All the processed images were then co-registered to an anatomical atlas and transformed into a matrix with an isotropic voxel resolution of 200 µm. The intersection of all rat brains was used to produce a brain mask using PMOD Software (Bruker BioSpin) from where the ventricles were excluded before exporting the data into MATLAB for further analysis (R2017b, The MathWorks, Inc., Natick, USA).

Clinical imaging - Post-processing

ADC maps were calculated from the diffusion weighted images using the clinical software syngo.via frontier (Siemens Healthineers). Realignment, motion correction and co-registration to a common atlas space was performed using MATLAB and SPM12 software [5]. Afterwards, the brain tissue was masked excluding the ventricles, analogous to the preclinical image post-processing.

Machine learning – Random Forest Classifier

Matlab's *TreeBagger* Random Forest implementation does not directly allow to set *max_depth* and *max_feature* parameters, and therefore these parameters were not optimized. While it is possible to set the '*MaxDepth*' parameter for the *fitctree* method, which *TreeBagger* uses for creating classification trees, it only applies to the special case when the Tall input arrays. This was not the case in this study. The fitctree method grows deep decision trees by default based on the *MinLeafSize*, or *MinParentSize* parameters. *MinLeafSize* was set to 1. The remaining parameters were kept to Matlab's defaults.

Noise filter

A median filter was applied on the clinical data in order to optimally compare the shape similarity between the predicted stroke regions and the human delineations using the Dice Similarity Coefficient (DSC) [6]. The filter size was chosen by gradually increasing the kernel dimensions starting at 2×2×2 voxels while quantifying the similarity to GT. A kernel volume of 8×8×8 voxels optimally reduced surrounding noise in the ML-prediction. We also studied the effects of median filtering on the metrics of success. All results are shown in Supplementary Fig. S5.

Visualization - Joint probability maps

The intuition behind joint probability maps was to visualize the average stroke volume – determined by ML as well as T2W and ADC thresholding – over all the rats for each group. This allows an intuitive and objective visualization of the entire stroke region in a single slide. To this end, we first selected the slices of interest (axial slice with the largest stroke extension and its two adjacent slices) for each rat based on the acute stroke probability maps obtained either via the optimal GMM or the trained RFC. Afterwards, the ML joint probability map was calculated by taking an average of all the coronal selected slices across all the rats of the respective group. Strokes produced by the MCAO model are consistently anatomically confined to the middle cerebral artery territory of the brain and so the joint probability maps provide a good representation of the stroke region. The thresholding joint probability maps were calculated based on the lesion classification maps obtained either through T2W or ADC

thresholding. However, for the sake of comparison, the slices of interest derived from ML were also used in calculating the thresholding joint probability maps.

Histology and Immunohistochemistry

All histological sections were stained with H&E and Luxol fast blue (LFB). Immunohistochemistry (IHC) was performed on an automated immunostainer (Ventana Medical Systems Inc, Roche Diagnostic, Mannheim, Germany) following the supplier's protocols for open procedures with slight modifications. All slides were stained with the antibody GFAP (anti-Glial Fibrillary Acidic Protein, Dakocytommation, Glostrup, Denmark). Appropriate positive and negative controls were used to confirm the adequacy of the staining.

Calculation of metrics

The following conditions and equations were used for calculating classification metrics:

$P = \text{Condition positive: number of real positive voxels in the data}$

$N = \text{Condition negative: number of real negative voxels in the data}$

$TP = \text{True positive}$

$TN = \text{True negative}$

$FP = \text{False positive}$

$FN = \text{False negative}$

$\text{Sensitivity} = TP/P$

$\text{Specificity} = TN/N$

$\text{Positive predictive value (PPV)} = TP/TP+FP$

$\text{Negative predictive value (NPV)} = TN/TN+FN$

$\text{Accuracy} = TP+TN/TP+TN+FP+FN$

$\text{Mathew's Correlation Coefficient (MCC)} =$

$(TPxTN–FPxFN)/ \surd((TP+FP)(TP+FN)(TN+FP)(TN+FN))$

$$Dice\ coefficient\ (DSC) = \frac{2|Segmentation\ Mask\ \cap Ground\ truth|}{|Segmentation\ Mask| + |Ground\ truth|}$$

Supplementary Data

Histological evaluations

We ascertained tissue characteristics of the stroke regions at 24 h and 1 week after stroke induction in order to further understand the imaging characteristics from MRI at the corresponding time points (Fig. 2 in main manuscript). All rats showed lesions in the ipsilateral hemisphere in the cortex and in the caudate/putamen with different sizes. After 24 h of middle cerebral artery occlusion the lesions were clearly visible as fresh ischemic infarctions with large edematous areas. Additionally, enlarged blood vessels were observed. In all animals the special stain LFB showed lack of axons in the stroke areas predominantly in the caudate/putamen. In association with the astrocyte-specific filament, the GFAP stain was negative in the stroke area at 24 h. Taken together, the 24 h time point evaluation showed a clear fresh infarct area with increased edema, but no clear evidence of neuroinflammation. At

the same time, Analysis of Variance (ANOVA) showed no significant difference between the stroke areas between the groups (neuroprotection), which was consistent with manually drawn ROIs.

At 1 week after stroke, histology showed smaller focalized lesions than the 24 h evaluations (Fig. 2A-B in main manuscript). H&E histology at 1 week clearly showed macrophages and astrocytes around and within the lesions, as well as neovascularization and starting fibrosis. We quantified the difference in area from 24 h to 1 week. The measurement of the stroke areas showed a 50% reduction over time, but these findings were not significantly different (Fig. 2B). Quantification using MRI however yielded a different result. We noticed during the analysis that the regions of interest produced by human experimenters at 24 h and the ground truth histology presented a volume mismatch (Fig. 3 and Fig. 4). We stipulated that this mismatch was produced by falsely classified stroke region, which presented edema and so we evaluated the T2-weighted signal intensity, a known imaging biomarker of vasogenic edema. There was significantly less edema at the 1-week time point in comparison to the findings in the 24 h histology (Fig. 2C). These data suggest edema may play a role in misclassification by humans, which the ML algorithm is able to discriminate using ADC. This was also evidenced using GFAP staining, which showed increased gliosis around the stroke area. This allowed a clearer visualization of the stroke core, clearly delimited in stark contrast to the diffuse and undistinguishable larger edematous areas of the 24 h timepoint. However, visually there was no clear difference in reactive gliosis between all groups at 1 week. Worth noting is that not all animals in the 1-week time point presented a strong reactive gliosis, which did not allow us to use it to quantify core regions to correlate with H&E measurements. At the same time, although LFB produces clear images of the white matter deterioration, it does not help identify the stroke core clearly. Taken together, histological analysis allowed us to confirm the evaluations we performed using MRI and allowed us to validate the stroke segmentation findings produced through our ML framework.

Supplementary Discussion

Probability maps

It can be noticed that the exemplary probability maps of the rats shown in Supplementary figure S3 mainly classify the stroke regions with a high probability (~0.95-1.0). The trained RF classifier behaves in such a manner due to two main reasons. First, the RF classifier was trained using the voxel-wise cluster labels derived from the optimal GMM and not the posterior probabilities. Using a classifier that takes into account the uncertainty of training labels (in order words can be trained on probabilistic labels) might alleviate this issue. Second, the classifier's task was simplified to a two-class classification problem (stroke/non-stroke), unlike the GMM clustering where the entire brain was divided into several clusters (5 in the case of optimal GMM). This resulted in a highly distinct signature of the stroke cluster, causing the trained model to classify test observations with high probability.

Normalization of Clinical datasets

Certain corrections play an important role in automated approaches, such as field strength differences between scanners, signal intensity corrections (resulting from different coils) and normalization of parameters. In this work, the normalization of the T2W parameter in the clinical subjects was vital for the successful model translation. However, this was not necessary for the animal data acquired in this study, mainly due to the standardized imaging protocols across the cohorts. Nonetheless, we suggest performing an instance-wise normalization in examinations where deviations in the T2W parameter across different animals are expected.

Noise Reduction

We were interested in the reduced DSC values of both 24 h and 1-week timepoints in humans, since visually there was seemingly good correspondence with the manually delineated stroke regions in the four patients. Scattered voxels not corresponding to the diagnosed focal stroke lesion played a role in the metric of similarity. In order to objectively explore the similitude of the predicted stroke area to estimated ground truth (EGT) without this noise, we systematically applied a median filter with increasing kernel volumes and calculated every corresponding metric (Supplementary figure S5). Median filtering with optimal kernel size (8×8×8 voxels) resulted in highest median similarity between the ML stroke region and the EGT. The removal of noise in the prediction maps also increased MCC and positive predictive value. The application of an 8^3 kernel led to a results similar as manual delineation, which was useful to recognize very small strokes (Supplementary figure S5). It must be pointed out that median-filter is an important tool to enhance model prediction and is normally used before data training. We applied the noise filtering after GMM and before RFC training, however the metrics in humans did not present the same improvement as applying the filter as a post-processing step.

Supplementary References

1. Longa EZ, Weinstein PR, Carlson S, Cummins R. Reversible middle cerebral artery occlusion without craniectomy in rats. Stroke. 1989; 20: 84–91.
2. Maier FC, Wehrl HF, Schmid AM, et al. Longitudinal PET-MRI reveals β-amyloid deposition and rCBF dynamics and connects vascular amyloidosis to quantitative loss of perfusion. Nat Med. 2014; 20: 1485–92.
3. Martirosian P, Klose U, Mader I, Schick F. FAIR true-FISP perfusion imaging of the kidneys. Magn Reson Med. 2004; 51: 353–61.
4. Castaneda Vega S, Weinl C, Calaminus C, et al. Characterization of a novel murine model for spontaneous hemorrhagic stroke using in vivo PET and MR multiparametric imaging. Neuroimage. 2017; 155: 245–56.
5. Penny W, Friston K, Ashburner J, Kiebel S, Nichols T. Statistical Parametric Mapping [Internet]. Elsevier; 2007.
6. Dice LR. Measures of the Amount of Ecologic Association Between Species. Ecology. 1945; 26: 297–302.

Supplementary Figure legends

Fig. S1. Experimental workflow. The above chart depicts the different post-processing of preclinical (yellow) and clinical data (black). Rat MRI data and human MRI data were acquired 24 h post-stroke onset with follow-up MRIs after 1-week (represented by the black boxes). The rat training dataset was used to identify a stroke cluster via Gaussian Mixture Modeling followed by training of a Random Forest Classifier (RFC). The trained RFC model was applied on the 24 h MRI dataset of Testing rats and on 24 h human MRIs. Regions of interest (ROI) were drawn on 24 h post-stroke MRI for humans and animals (blue). MRI ROIs of the 1-week follow-up were used as estimated ground truth (EGT) for rats and humans correspondingly. Histology was performed at 24 h on a subset of 9 animals while another larger group of 20 animals was evaluated with histology after 1 week (brown). ROI analysis was also produced on the 24 and 1-week histology animal data. Metrics of prediction success and correlations to final stroke volume were calculated on all data by comparing against Manual ROIs at 24h or EGT. Finally, a modified variant of the rat-trained RFC was applied on the clinical data to assess the feasibility of feature translation and stroke identification in humans.

Fig. S2. Percentage of predicted stroke voxels in non-stroke hemisphere. The plot depicts the fraction of voxels in the contralateral hemispheres of all the Training group rats that were labeled as stroke by Gaussian mixture model (GMM) with varying number of mixture components. Since the contralateral hemispeheres did not present stroke lesions, the optimal number of mixture components was detemined based on a GMM configuration that resulted in a reduced contralateral stroke fraction (less than 1%, highlighted in yellow). Any further increase in the number of mixture components subdivided the stroke cluster.

Fig. S3. Examples of single animal comparisons per group. Training, Test-group1 and Test-group2 examples depicting coronal rat brain apparent diffusion coefficient (ADC) and T2-weighted (T2W) images along with the machine learning prediction as a probability map. The exemplary cases show the role that the interaction between both the parameters plays in predicting stroke regions in the probability maps. The Training group example in the first row shows a stroke delimited to the left hemisphere with reduced ADC and increased signal intensity in the T2W image. The right column shows the segmented ischemic stroke region. The second row shows a similar pattern in ADC and T2W imaging, however here, the ADC shows hyperintensities in the left stroke hemisphere and strong hypointensity artifacts (red arrow) on the contralateral hemisphere. This type of artifact is common in echo planar imaging acquisitions. The lack of T2W hyperintensity and the strong uncharacteristic ADC reduction in the artifact aids the correct classification in the probability map. The bottom row presents a rat example from the Test-group2, which shows a stroke region similar to the Training group rat, however ADC shows a folding artifact on the cortex produced by the echo planar image that leads to ADC values within the range of the stroke cluster. The algorithm misclassifies in this case these small amount of voxels and produces false positives, denoting how artifacts may impact the method.

Fig. S4. Quantification of stroke volume using all animals at 24 h. The boxplots show the distribution of the stroke volume calculated by the apparent diffusion coefficient and T2-weighted image thresholding (ADCth and T2Wth) methods, as well as the manual region of interest and the machine learning (ML) calculations. ANOVA showed a significant difference between the methods for the Training n=12, $F(3,44)=14.8$, $P<0.001$ and Testing group (n=20), $F(3,76)=21.2$, $P<0.001$, demonstrating significant overestimation by the thresholding approaches against ML ($P<0.001$). The human and the ML aproaches were not significantly different. The ML model allowed an objective and automatized evaluation of therapy induced changes in stroke volume. The boxplot presents several metrics in symbols: Mean (+), median

(red line), 1st quartile of the data (box upper border), 3rd quartile of the data (box lower border) and 15% outmost border of the dataset (whisker). * = P<0.05, ** = P<0.01.

Fig. S5. Effect of the median-filter's kernel size on clinical metrics at 1-week.

A. The boxplots show different metrics as a function of the median filter's kernel size when comparing the machine learning algorithm's (ML) predictions to the ground truth (EGT). On the horizontal axis, the raw ML prediction metric is plotted next to increasing median filter volumes (the kernel size is isotropic in all three directions to the labeled values). Median Dice Coefficient (DSC), Matthew's correlation coefficient (MCC) and positive predictive value (PPV) present an improvement as the noise is removed by the filter compared to their raw scores. The boxplots present several measures in symbols: Mean (+), median (red line), 1st quartile of the data (box upper border), 3rd quartile of the data (box lower border) and 15% outmost border of the dataset (whisker). **B**. The image on top left shows the raw machine learning prediction (Raw ML) for patient 2 from the stroke patients group. To the right, the same prediction has been processed using a median filter with increasing kernel volumes shown from left to right. As can be seen in the image, a kernel of dimensions 16×16×16 reshaped and reduced similarity between the stroke volume predictions the manual 24 h and 1-week delineations. The graph on the bottom right shows the size of the kernel relative to the volume of the brain. The bar in yellow shows the percent of the brain represented by the delineated stroke volume at 24 h. The black bars depict the volumes of the different kernels illustrating the relationship of the median filter volume to that of the small stroke lesion (red arrow) in the bottom. The 8×8×8 kernel's volume is slightly smaller than the small stroke lesion in Patient 2 and therefore it may have small, but measurable effects in determining the shape of the stroke.

Fig. S7. Metrics of stroke prediction on clinical data. The metrics calculated correspond to the four radiologically confirmed stroke cases. Here we compared the machine learning (ML) prediction to the manual 1-week timepoint delineation by the radiologist or ground truth (GT) and the manual delineation by the radiologist at 24 h (Man). Accuracy, Specificity and negative predictive value were remarkably high in all comparisons. Dice similarity coefficient (DSC) was highest between ML and Man at 24 h. Manual delineation presented a similar shape as ML, when compared to GT. Matthew's Correlation Coefficient (MCC) results were similar to the DSC. The median sensitivity and positive predictive value was similar between all comparisons. The boxplots present several measures in symbols: Mean (+), median (red line), 1st quartile of the data (box upper border), 3rd quartile of the data (box lower border) and 15% outmost border of the dataset (whisker).

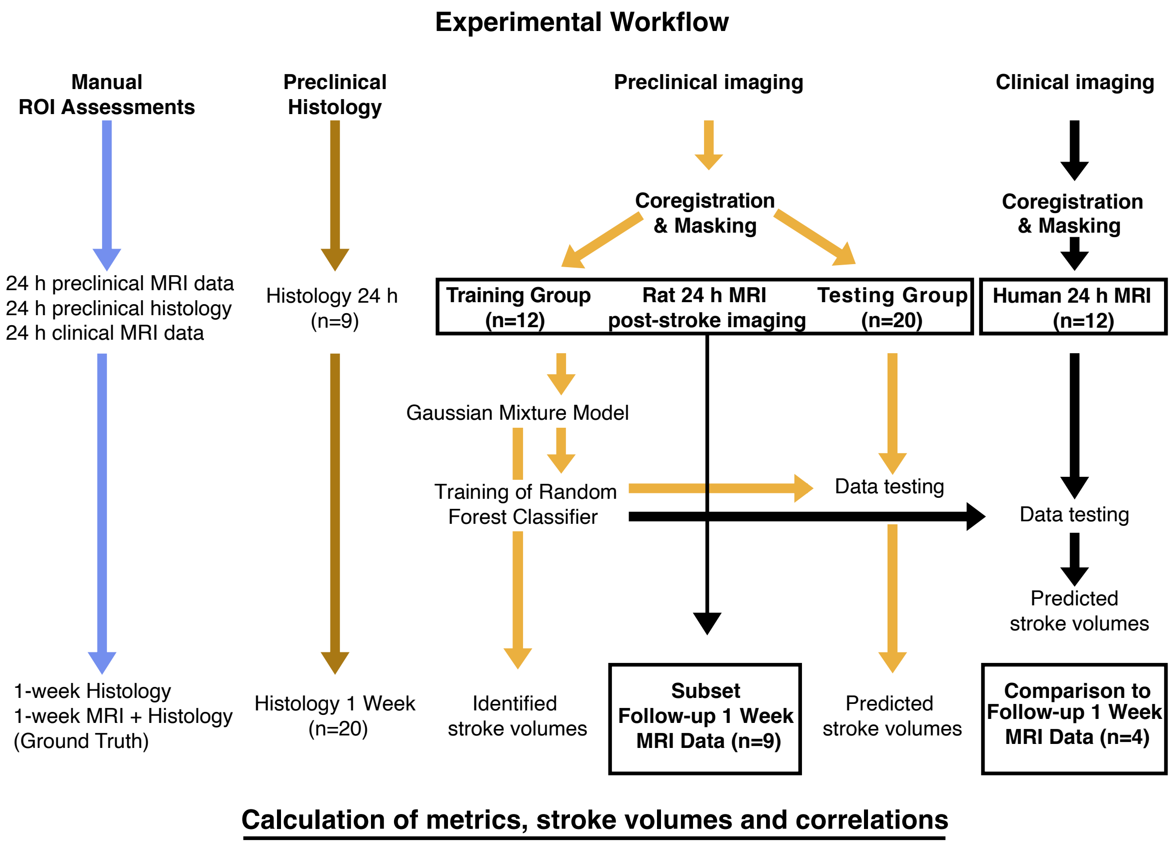

Supplementary Figure S1

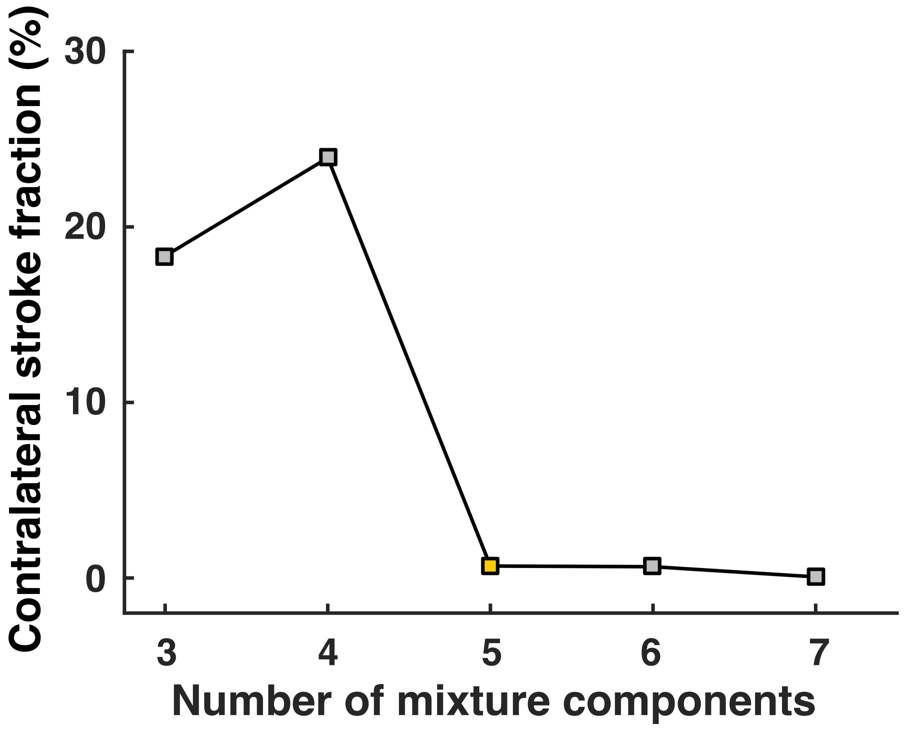

Supplementary Figure S2

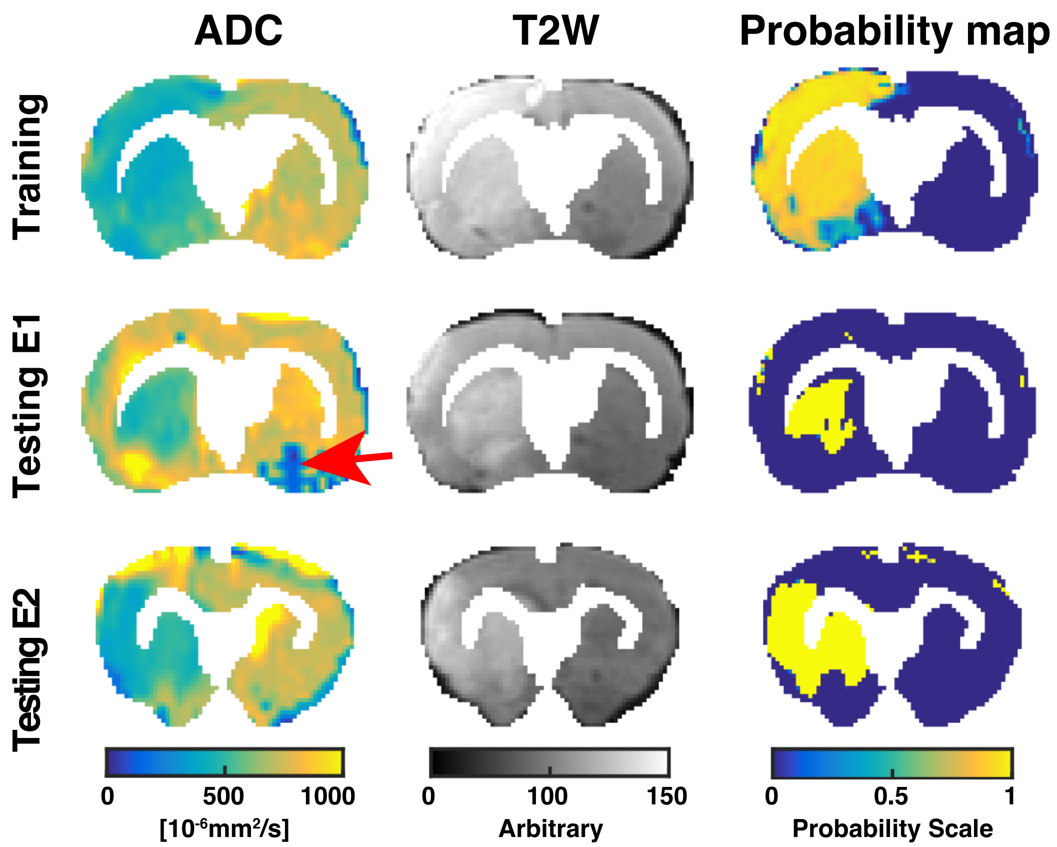

Supplementary Figure S3

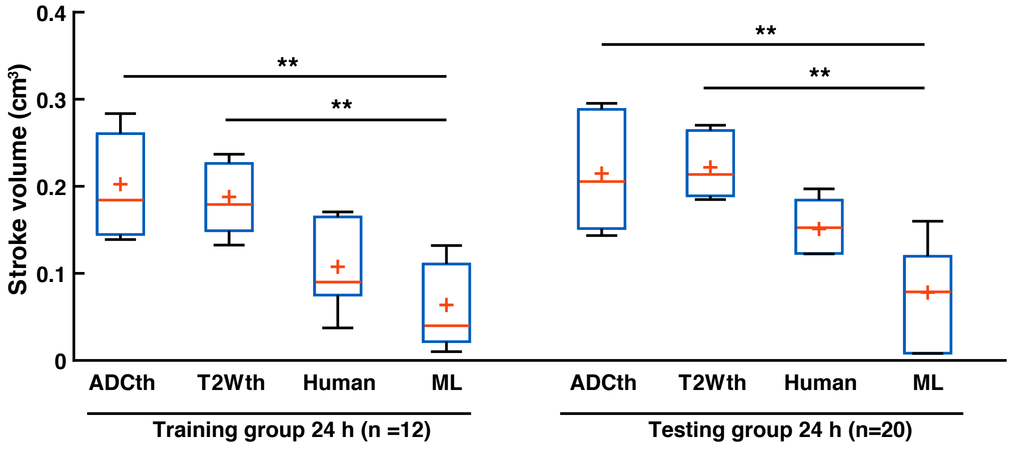

Supplementary Figure S4

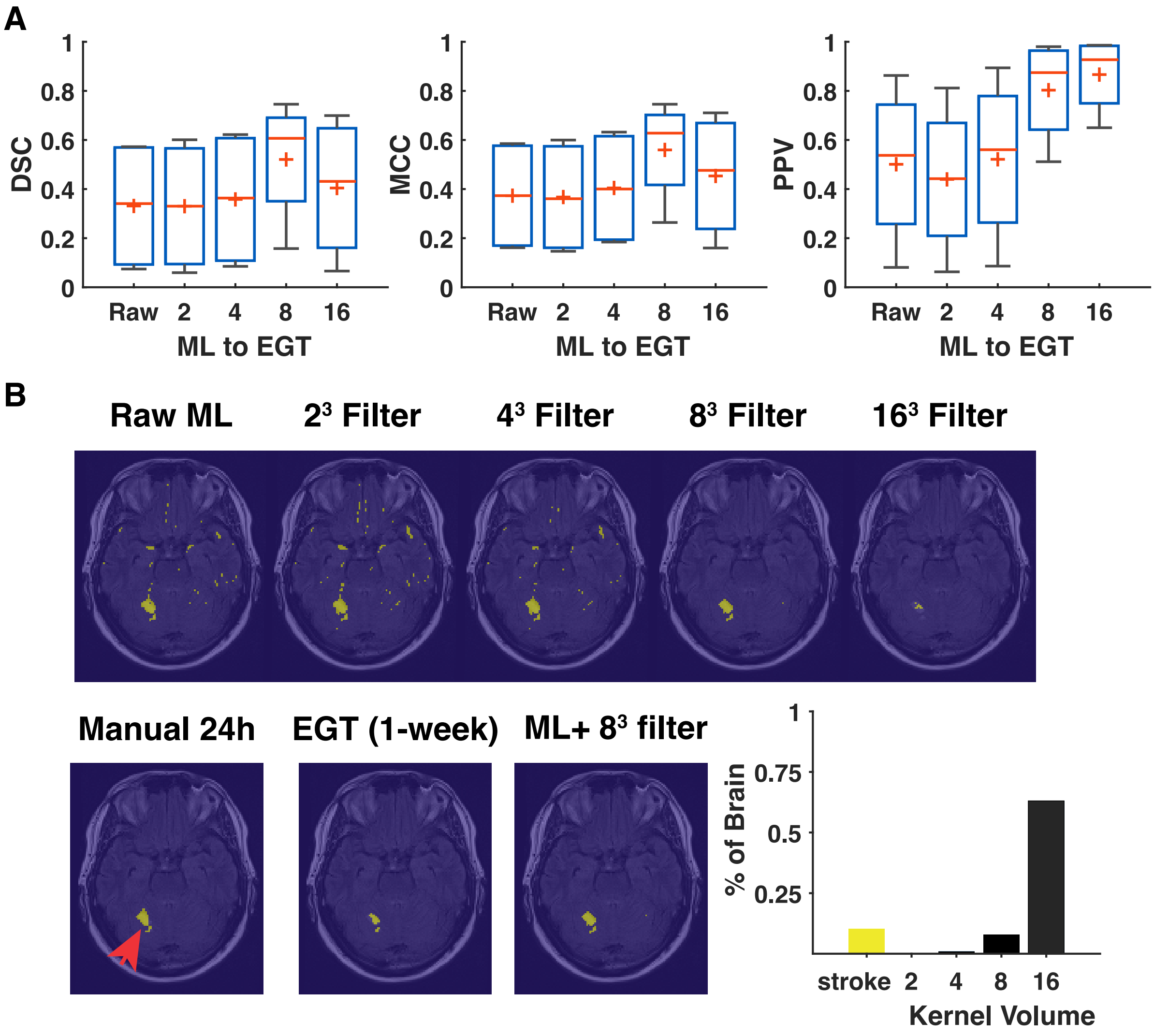

Supplementary Figure S5

	Training	Testing	Total
Rat 24 h MRI	n=12	n=20	n=32
Rat 24 h Histology	n=2	n=7	n=9
Rat 1-week MRI	n=3	n=6	n=9
Rat 1-week Histology	n=8	n=12	n=20
Human 24 h MRI	-	n=8	n=8
Human 1-week MRI	-	n=4	n=4

Table 1. Distribution and number of subjects per group.

Parameter	Groups compared		Lower confidence interval	Estimate	Upper confidence interval	P-value
Dice coefficient	ADCth	T2wTh	-0.428	-0.081	0.264	1
	ADCth	Manual	-0.480	-0.133	0.212	1
	ADCth	ML	-0.335	0.010	0.356	1
	T2wTh	Manual	-0.398	-0.051	0.294	1
	T2wTh	ML	-0.254	0.092	0.438	1
	Manual	ML	-0.202	0.144	0.490	1
Accuracy	ADCth	T2wTh	-0.096	-0.037	0.021	0.514796
	ADCth	Manual	-0.128	-0.069	-0.009	0.0149418
	ADCth	ML	-0.119	-0.059	-0.001	0.0463073
	T2wTh	Manual	-0.091	-0.031	0.027	0.8450172
	T2wTh	ML	-0.081	-0.022	0.036	1
	Manual	ML	-0.049	0.009	0.068	1
Matthew's correlation coefficient	ADCth	T2wTh	-0.451	-0.119	0.211	1
	ADCth	Manual	-0.500	-0.168	0.162	0.9701596
	ADCth	ML	-0.353	-0.021	0.309	1
	T2wTh	Manual	-0.380	-0.049	0.282	1
	T2wTh	ML	-0.233	0.097	0.429	1
	Manual	ML	-0.184	0.147	0.478	1
Sensitivity	ADCth	T2wTh	-0.446	-0.103	0.239	1
	ADCth	Manual	-0.426	-0.083	0.259	1
	ADCth	ML	-0.140	0.202	0.545	0.3070577
	T2wTh	Manual	-0.323	0.019	0.362	1
	T2wTh	ML	-0.037	0.305	0.648	0.0266207
	Manual	ML	-0.056	0.285	0.628	0.0440178
Positive predictive value	ADCth	T2wTh	-0.454	-0.101	0.250	1
	ADCth	Manual	-0.520	-0.168	0.183	1
	ADCth	ML	-0.525	-0.173	0.179	1
	T2wTh	Manual	-0.418	-0.066	0.285	1
	T2wTh	ML	-0.423	-0.071	0.281	1
	Manual	ML	-0.357	-0.004	0.347	1
False discovery rate	ADCth	T2wTh	-0.250	0.101	0.454	1
	ADCth	Manual	-0.183	0.168	0.520	1
	ADCth	ML	-0.179	0.173	0.525	1
	T2wTh	Manual	-0.285	0.066	0.418	1
	T2wTh	ML	-0.281	0.071	0.423	1
	Manual	ML	-0.347	0.004	0.357	1
Negative predictive value	ADCth	T2wTh	-0.045	-0.015	0.014	0.9650712
	ADCth	Manual	-0.041	-0.011	0.018	1
	ADCth	ML	0.004	0.034	0.064	0.0168658
	T2wTh	Manual	-0.025	0.004	0.034	1
	T2wTh	ML	0.019	0.049	0.079	0.0003082
	Manual	ML	0.015	0.045	0.075	0.0009567
Specificity	ADCth	T2wTh	-0.094	-0.037	0.019	0.4355951
	ADCth	Manual	-0.134	-0.077	-0.020	0.0036188
	ADCth	ML	-0.167	-0.110	-0.053	3.163E-05
	T2wTh	Manual	-0.096	-0.039	0.017	0.3608099
	T2wTh	ML	-0.129	-0.072	-0.015	0.0063794
	Manual	ML	-0.090	-0.033	0.023	0.651647
False positive rate	ADCth	T2wTh	-0.019	0.037	0.094	0.4355951
	ADCth	Manual	0.020	0.077	0.134	0.0036188
	ADCth	ML	0.053	0.110	0.167	3.163E-05
	T2wTh	Manual	-0.017	0.039	0.096	0.3608099
	T2wTh	ML	0.015	0.072	0.129	0.0063794
	Manual	ML	-0.023	0.033	0.090	0.651647

Table 2. Confidence intervals and P-values of multiple comparisons of preclinical metrics at 1-week (Corresponding to Figure 6 in main manuscript).

Metric	Df	Df-error	F-Value	P-Value
Dice Coefficient	2	21	5.64	0.01
Accuracy	2	21	13.35	2.0e-04
Mathew's Correlation coefficient	2	21	7.84	3.0e-04
Sensitivity	2	21	11.7	4.00e-04
Positive predictive value	2	21	11.0	5.00e-04
False discovery rate	2	21	11.0	5.00e-04
Negative predictive value	2	21	0.45	0.64
Specificity	2	21	21.3	8.96e-04
False positive rate	2	21	21.3	8.96e-04

Table 3. Human metrics ANOVA statistics 24 h

Parameter	Groups compared		Lower confidence interval	Estimate	Upper confidence interval	P-value
Dice coefficient	ADCth	T2Wth	-0,136	0,068	0,272	1
	ADCth	ML	-0,390	-0,186	0,017	0,081075635
	T2Wth	ML	-0,458	-0,254	-0,050	0,011657912
Accuracy	ADCth	T2Wth	-0,050	-0,002	0,045	1
	ADCth	ML	-0,1320	-0,083	-0,035	0,000541599
	T2Wth	ML	-0,129	-0,081	-0,033	0,000729721
Matthew's correlation coefficient	ADCth	T2Wth	-0,053	0,119	0,292	0,25827889
	ADCth	ML	-0,315	-0,143	0,029	0,128823339
	T2Wth	ML	-0,435	-0,262	-0,089	0,002166707
Sensitivity	ADCth	T2Wth	0,114	0,339	0,564	0,002339902
	ADCth	ML	0,156	0,381	0,606	0,000738323
	T2Wth	ML	-0,183	0,041	0,266	1
Positive predictive value	ADCth	T2Wth	-0,203	0,037	0,278	1
	ADCth	ML	-0,596	-0,355	-0,115	0,002819615
	T2Wth	ML	-0,634	-0,393	-0,152	0,001063348
False discovery rate	ADCth	T2Wth	-0,278	-0,037	0,203	1
	ADCth	ML	0,115	0,355	0,596	0,002819615
	T2Wth	ML	0,152	0,393	0,634	0,001063348
Negative preditive value	ADCth	T2Wth	-0,019	0,009	0,037	1
	ADCth	ML	-0,019	0,008	0,037	1
	T2Wth	ML	-0,029	-0,001	0,027	1
Specificity	ADCth	T2Wth	-0,052	-0,010	0,031	1
	ADCth	ML	-0,138	-0,096	-0,054	1,9543E-05
	T2Wth	ML	-0,1271	-0,085	-0,043	9,38755E-05
False positive rate	ADCth	T2Wth	-0,031	0,010	0,052	1
	ADCth	ML	0,054	0,096	0,138	1,9543E-05
	T2Wth	ML	0,043	0,085	0,127	9,38755E-05

Table 4. Confidence intervals and P-values of multiple comparisons of clinical metrics at 24 h (Corresponding to Figure 8 in main manuscript).

Metric	Df	Df-error	F-Value	P-Value
Dice Coefficient	3	12	2.41	0.117
Accuracy	3	12	12.0	6.0e-03
Mathew's Correlation coefficient	3	12	4.05	0.03
Sensitivity	3	12	0.35	0.79
Positive predictive value	3	12	4.97	0.018
False discovery rate	3	12	4.97	0.018
Negative predictive value	3	12	0.11	0.954
Specificity	3	12	24.6	2.07e-05
False positive rate	3	12	24.6	2.07e-05

Table 5. Human metrics ANOVA statistics 1-week.

Parameter	Groups compared		Lower confidence interval	Estimate	Upper confidence interval	P-value
Dice coefficient	ADCth	T2Wth	-0,520	-0,031	0,457	1
	ADCth	Manual	-0,864	-0,375	0,113	0,193222177
	ADCth	ML	-0,630	-0,141	0,347	1
	T2Wth	Manual	-0,833	-0,344	0,144	0,278417382
	T2Wth	ML	-0,599	-0,110	0,378	1
	Manual	ML	-0,255	0,233	0,722	0,943781655
Accuracy	ADCth	T2Wth	-0,056	0,017	0,091	1
	ADCth	Manual	-0,170	-0,096	-0,022	0,008912929
	ADCth	ML	-0,157	-0,083	-0,009	0,02413076
	T2Wth	Manual	-0,187	-0,113	-0,039	0,002469558
	T2Wth	ML	-0,174	-0,100	-0,026	0,00639019
	Manual	ML	-0,061	0,012	0,086	1
Matthew's correlation coefficient	ADCth	T2Wth	-0,468	-0,048	0,370	1
	ADCth	Manual	-0,840	-0,421	-0,002	0,048362632
	ADCth	ML	-0,612	-0,193	0,225	1
	T2Wth	Manual	-0,791	-0,372	0,0466	0,095881621
	T2Wth	ML	-0,563	-0,144	0,274	1
	Manual	ML	-0,191	0,228	0,647	0,671931392
Sensitivity	ADCth	T2Wth	-0,843	-0,139	0,564	1
	ADCth	Manual	-0,732	-0,028	0,675	1
	ADCth	ML	-0,618	0,0856	0,789	1
	T2Wth	Manual	-0,593	0,110	0,814	1
	T2Wth	ML	-0,478	0,225	0,928	1
	Manual	ML	-0,589	0,114	0,818	1
Positive predictive value	ADCth	T2Wth	-0,599	-0,022	0,554	1
	ADCth	Manual	-1,180	-0,603	-0,026	0,038181176
	ADCth	ML	-0,930	-0,3538	0,223	0,462754197
	T2Wth	Manual	-1,157	-0,580	-0,003	0,048025265
	T2Wth	ML	-0,908	-0,331	0,245	0,572639187
	Manual	ML	-0,327	0,249	0,826	1
False discovery rate	ADCth	T2Wth	-0,554	0,022	0,599	1
	ADCth	Manual	0,026	0,603	1,180	0,038181176
	ADCth	ML	-0,223	0,353	0,930	0,462754197
	T2Wth	Manual	0,003	0,580	1,157	0,048025265
	T2Wth	ML	-0,245	0,331	0,908	0,572639187
	Manual	ML	-0,826	-0,249	0,327	1
Negative preditive value	ADCth	T2Wth	-0,085	-0,011	0,062	1
	ADCth	Manual	-0,083	-0,008	0,065	1
	ADCth	ML	-0,076	-0,002	0,071	1
	T2Wth	Manual	-0,071	0,002	0,076	1
	T2Wth	ML	-0,064	0,009	0,083	1
	Manual	ML	-0,067	0,006	0,080	1
Specificity	ADCth	T2Wth	-0,028	0,027	0,083	0,886152301
	ADCth	Manual	-0,151	-0,095	-0,039	0,001013575
	ADCth	ML	-0,145	-0,089	-0,033	0,001807937
	T2Wth	Manual	-0,179	-0,123	-0,067	9,70747E-05
	T2Wth	ML	-0,173	-0,116	-0,060	0,00016065
	Manual	ML	-0,049	0,006	0,062	1
False positive rate	ADCth	T2Wth	-0,083	-0,027	0,028	0,886152301
	ADCth	Manual	0,039	0,095	0,151	0,001013575
	ADCth	ML	0,033	0,089	0,145	0,001807937
	T2Wth	Manual	0,067	0,123	0,179	9,70747E-05
	T2Wth	ML	0,060	0,116	0,173	0,00016065
	Manual	ML	-0,062	-0,006	0,049	1

Table 6. Confidence intervals and P-values of multiple comparisons of preclinical metrics at 1-week (Corresponding to Figure 9 in main manuscript).
